# Serum vaccine antibody concentrations in adults exposed to per- and polyfluoroalkyl substances: A birth cohort in the Faroe Islands

**DOI:** 10.1080/1547691X.2021.1922957

**Published:** 2021-12

**Authors:** Yu-Hsuan Shih, Annelise J. Blomberg, Marie-Abèle Bind, Dorte Holm, Flemming Nielsen, Carsten Heilmann, Pál Weihe, Philippe Grandjean

**Affiliations:** a Department of Environmental Health, Harvard T. H. Chan School of Public Health, Cambridge, MA, USA; b Department of Medicine, Massachusetts General Hospital and Harvard Medical School, Boston, MA, USA; c Department of Clinical Immunology, Odense University Hospital, Odense, Denmark; d Department of Environmental Medicine, University of Southern Denmark, Odense, Denmark; e Pediatric Clinic, National University Hospital, Copenhagen, Denmark; f Centre of Health Science, Faculty of Health Sciences, University of the Faroe Islands, Torshavn, Faroe Islands; g Department of Occupational Medicine and Public Health, The Faroese Hospital System, Torshavn, Faroe Islands

**Keywords:** Per- and polyfluoroalkyl substances, PFAS, vaccine response, immune function, antibodies, prospective study

## Abstract

Per- and polyfluoroalkyl substances (PFASs) are highly persistent in the environment and may cause depressed immune function. Previous studies have linked PFAS exposure to lower vaccine responses in children, but research in adults is limited. Therefore, the present study evaluated the associations between exposure to PFASs and serum antibody concentrations in adults vaccinated at age 28 years in the Faroe Islands. PFAS concentrations were determined from cord-blood collected at birth and serum samples collected at ages 7, 14, 22, and 28 years. Serum antibody concentrations against hepatitis type A and B, diphtheria, and tetanus were analyzed from blood samples collected about 6 mo after the first vaccine inoculation at age 28 years. Linear regression models were used to estimate changes in antibody concentration for each doubling of PFAS concentration. Potential effect modification by sex was assessed by including an interaction term between PFAS and sex. Although the 95% confidence intervals contain the null value, inverse trends were observed between serum perfluorooctanoate (PFOA) at ages 14 and 28 years and hepatitis type A antibody (anti-HAV) concentrations, as revealed by an estimated decrease of 0.71 (95% CI: −1.52, 0.09) and 0.24 (95% CI: −0.59, 0.10) signal-to-cutoff ratio for each doubling of exposure, respectively. Inverse trends were also observed between serum PFOA at ages 22 and 28 years and hepatitis type B antibody (anti-HBs) concentration, with an estimated decrease of 21% (95% CI: −42.20%, 7.34%) and of 17% (95% CI: −35.47%, 7.35%) in anti-HBs for each doubling of exposure, respectively. Sex-specific associations with anti-HAV were observed for cord-blood PFASs and serum PFAS concentrations at ages 7 and 14 years. No inverse associations of PFAS exposure were found with diphtheria and tetanus antibody concentrations. Future studies are needed to confirm these findings and further investigate the effects of PFASs on adult immune function.

## Introduction

Per- and polyfluoroalkyl substances (PFASs) are synthetic fluorinated compounds. Given their hydrophobic and oleophobic properties, they have been widely produced since the 1940s. Some PFASs are highly persistent organic pollutants and thus ubiquitous in the environment, with human exposure occurring through dietary intake, ingestion of contaminated water and dust, and inhalation of contaminated air (Nakayama et al. 2019; Sunderland et al. 2019).

Exposure to PFASs has been associated with immunotoxicity in experimental *in vitro* and animal models as well as epidemiologic studies (DeWitt et al. 2019). The European Food Safety Authority (EFSA) recently concluded that immunotoxicity should be considered as a critical effect of the major PFASs and that tolerable exposure limits should be based on preventing such adverse effects (Schrenk et al. 2020). Previous studies of children and adolescents have associated PFASs with increased risk of immune-related health outcomes (e.g. common colds, fever, eczema, asthma, and atopic dermatitis) (Granum et al. 2013; Dalsager et al. 2016; Goudarzi et al. 2017; Timmermann et al. 2017; Chen et al. 2018; Averina et al. 2019; Impinen et al. 2019; Ait Bamai et al. 2020; Huang et al. 2020; Kvalem et al. 2020) and decreased vaccine responses (Grandjean et al. 2012, Granum et al. 2013; Mogensen et al. 2015; Kielsen et al. 2016; Stein et al. 2016; Grandjean et al. 2017a, 2017b; Zeng et al. 2019; Timmermann et al. 2020; Dalsager et al. 2021).

There is relatively little epidemiologic research evaluating the effects of PFAS exposure on immune function in adult populations, and existing results have been equivocal. Three cross-sectional studies in the United States and China have found PFAS exposures to be associated with reduced antibody levels against rubella, hepatitis type B, and influenza A H3N2 in adults (Looker et al. 2014; Pilkerton et al. 2018; Zeng et al. 2020). Another study in a U.S. adult population found no evidence of a relationship between PFASs and influenza A H1N1 antibody response (Stein et al. 2016). Previous studies have examined the effects of PFASs on immune-related health outcomes (e.g. cold and flu infections, ulcerative colitis, or inflammatory bowel disease) in adults and reported discordant results (Steenland et al. 2013, 2015; Looker et al. 2014; Xu et al. 2020); associations between PFASs and decreased levels of proteomic markers, such as tumor necrosis factor (TNF)-α and various interleukins (IL), have been reported (Bassler et al. 2019; Salihovic et al. 2020). However, the majority of the existing epidemiologic studies are cross-sectional in nature without considering higher burden of PFASs in children that may potentially affect health later in life (Kato et al. 2011; Mondal et al. 2012).

Thus, the longitudinal study reported here aimed to evaluate the associations of cord-blood PFAS concentrations and serum PFAS concentrations measured at ages 7, 14, 22, and 28 years with vaccine antibody responses at age 28 years in a prospective birth cohort in the Faroe Islands (Cohort 1), born in 1986–1987. Additionally, since there are known sex differences in PFAS exposure patterns and immune responses (Harada et al. 2004; Klein and Flanagan 2016), potential sex-dependent associations of PFASs were also estimated.

## Materials and methods

### Study population

**Figure 1.**
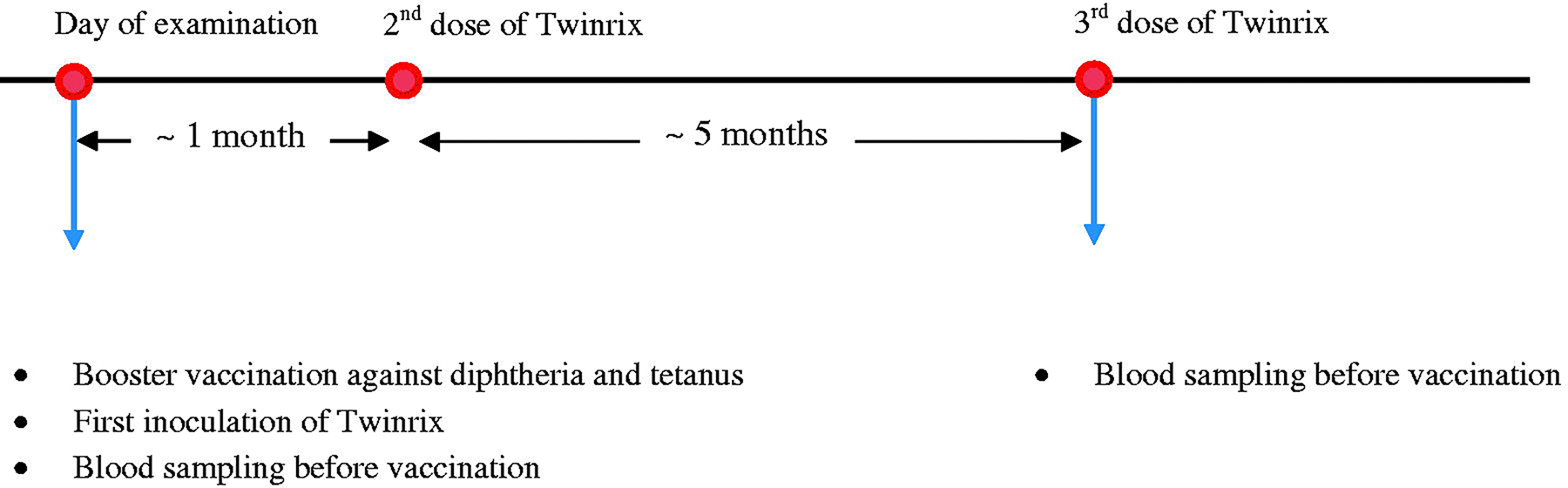
Vaccination and blood sampling schedule.

The current study population is a subset of a birth cohort (*N* = 1,022) recruited from the three Faroese hospitals from 1986 to 1987 (Grandjean et al. 1992). Subjects enrolled in this cohort were singleton births born at or close to term. A cord-blood sample was collected at birth. Follow-up clinical examinations, including standard questionnaires and blood sample collection, were conducted among 90% of the cohort members at age 7 years, 87% at age 14 years, and 84% at age 22 years. In 2014 and 2015, all cohort members (96% eligible) were invited back for a 28-year examination that included an optional vaccination trial. The vaccination and blood sampling schedule is shown in Figure 1. A booster vaccination against diphtheria and tetanus and vaccination against hepatitis type A and B (Twinrix) were offered. Standard Twinrix administration includes three doses; the second dose 1 mo after the first, and the final dose 5 mo after the second. Diphtheria and tetanus booster vaccines were given on the same day as the first Twinrix dose. A baseline blood sample was collected on the day of the first dose, and a follow-up sample was collected when the subjects returned for the last dose of Twinrix. Both blood samples were obtained immediately preceding vaccine administration. Subjects who were vaccinated against diphtheria and tetanus only returned for follow-up blood sample collection about 6 mo after the first visit (*n* = 125; median: 183 days).

A total of 454 cohort members completed the full vaccination course of Twinrix without deviations from the intended schedule. Of these, 53 subjects were excluded who had previously been vaccinated for hepatitis type A or B. One subject was further excluded due to missing data on antibody concentrations, because blood sample was lost. Figure S1 shows the process for arriving at the final sample size for hepatitis type A and B analyses.

A total of 593 cohort members were re-vaccinated for diphtheria and tetanus; of these, 75 subjects who did not participate in the follow-up examination were excluded. Since there was no access to the information regarding any booster doses against diphtheria and tetanus after the routine vaccination at age 5 years, the following inclusion/exclusion criteria were applied. A total of 137 subjects were excluded whose diphtheria and tetanus antibody concentrations at age 7 were lower than the baseline concentration at age 28 (255 with available 7-year measurements). For the 263 subjects without antibody measures at age 7 years, 98 with baseline antibody concentrations of diphtheria ≥ 0.5 IU/mL or tetanus ≥ 0.2 IU/mL at age 28 years were excluded. These two cutoff points were determined based on distributions of baseline antibody concentrations of diphtheria and tetanus at age 28 years among 118 subjects that had baseline concentration at age 28 years lower than concentrations at age 7. Of the remaining 283 subjects, a further two subjects whose base-line antibody concentrations of diphtheria or tetanus were higher than the follow-up antibody concentrations were excluded. Figure S2 presents the sample selection process for diphtheria and tetanus analysis.

The study protocol was approved by the Faroese ethical review committee and the Harvard T.H. Chan School of Public Health institutional review board. Written informed consent was obtained from all participants.

### Assessing PFAS exposure

Concentrations of five major PFAS, e.g. perfluorooctane sulfonic acid (PFOS), per-fluorooctanoic acid (PFOA), perfluorohexane sulfonic acid (PFHxS), perfluorononanoic acid (PFNA), and perfluorodecanoic acid (PFDA), in samples of umbilical cord-blood and serum collected at ages 7, 14, 22, and 28 years (baseline blood sample) were determined using on-line solid-phase extraction followed by high performance liquid chromatography with tandem mass spectrometry (LC-MS/MS) (Haug et al. 2009). High measurement accuracy was suggested based on a within-batch coefficients of variation (CV) < 3%, and a between-batch CV < 5.2%. The limit of detection (LOD) was 0.05 ng/ml for cord-blood PFOA, PFHxS, and PFNA; the LOD was 0.03 ng/ml for all other PFASs. Only one participant had PFHxS concentrations at age 14 years below the LOD, and a value of 0.015 ng/ml was assigned.

### Assessing serum antibody concentration

Serum concentrations of anti-hepatitis A virus immunoglobulin (anti-HAV IgG) and hepatitis B surface antibody (anti-HBs) were analyzed from the follow-up blood sample using the Architect system (Abbott Diagnostics, Delkenheim, Germany). The Architect HAVAb IgG assay and Architect Anti-HBs assay are both two-step immunoassays using chemiluminescent micro-particle immunoassay (CMIA) technology. Specifically, the concentration of anti-HAV IgG was obtained by comparing the chemiluminescent signal in the reaction to the cutoff signal (S/CO ratio) determined from a calibration curve. Samples with S/CO values ≥ 1.0 were considered reactive and samples with S/CO < 1.0 were considered non-reactive. The concentration of anti-HBs was determined using a previously-generated Architect anti-HBs calibration curve. Samples with values ≥ 10 mIU/mL were considered reactive and those < 10 mIU/ml were considered non-reactive. Subjects with extreme anti-HBs concentrations (> 1,000 mIU/ml) were excluded from the subsequent analyses.

Serum concentrations of IgG antibodies against diphtheria and tetanus toxoids were measured from both the baseline and follow-up blood samples by the Statens Serum Institute (SSI; Copenhagen, Denmark). Baseline serum antibody concentrations were analyzed using a standard Vero cell-based neutralization assay (diphtheria) and an enzyme-linked immunosorbent assay using 2-fold dilutions of the serum (tetanus), as in earlier studies (Miyamura et al. 1974; Hendriksen et al. 1988). Calibration was conducted using both international and local standard antitoxins. The follow-up serum antibody concentrations were analyzed using Binding Site vaccine response assays (Binding Site; Birmingham, UK), following manufacturer instructions. Samples with antibody concentrations ≥ 0.1 IU/mL were considered as being protective against diphtheria or tetanus infections.

### Assessing covariates

Participant characteristics including sex, smoking status (current, ever, never) and current alcohol consumption (yes, no) were available from questionnaires. Height and weight were measured during physical examinations at ages 14, 22, and 28 years, and body mass index (BMI in kg/m^2^) was calculated. Obstetric information, including maternal age, gestational age, parity, and pre-pregnancy height and weight was abstracted from medical records (Grandjean et al. 1997). Concentrations of 14 polychlorinated biphenyls (PCB) congeners were measured from cord-blood as well as serum samples collected at ages 7, 14, 22, and 28 years using a gas chromatograph equipped with a dual-column and two micro-electron capture detectors (Agilent Santa Clara, CA). Total PCB (ΣPCB; μg/g lipid) was calculated by multiplying the sum of three individual congeners (i.e. PCB-138, PCB-153, and PCB-180) by two (Authority EEFS 2005).

### Statistical analyses

Characteristics of cohort members included in the hepatitis type A and B analyses as well as diphtheria and tetanus analyses overall and by sex are reported. Differences in antibody concentrations and PFAS concentrations by sex were evaluated using a Wilcoxon rank-sum test. Spearman’s rank correlation coefficients were used to assess pair-wise correlations among the PFAS concentrations.

Linear regression models were used to estimate the associations of PFAS concentrations, measured at different timepoints, with antibody concentrations for each PFAS-antibody association of interest. PFAS concentrations were modeled as log_2_-transformed continuous variables given its skewed distributions. Anti-HAV was modeled as an untransformed continuous variable since its distribution was normal, while anti-HBs, anti-diphtheria, and anti-tetanus were modeled as log_2_-transformed continuous variables due to their skewed distributions. β-Coefficients were expressed as percent change in antibody concentrations (S/CO changes for anti-HAV) per doubling of the PFAS exposure. The baseline antibody concentration of diphtheria or tetanus was also included as a covariate in models where the dependent variables were the concentration of diphtheria or tetanus at follow-up. All analyses were adjusted for sex. Potential effect modification by sex were also assessed by including a cross-product term between sex and the exposure of interest. The corresponding value of the observed Wald test statistic was used to obtain a *p*-value for interaction (*p*_*interaction*_). A *p*_*interaction*_ < 0.05 was considered significant for interaction effects.

Four sensitivity analyses were conducted to assess the robustness of the study findings. A generalized additive models (GAMs) was first conduction with penalized thin-plate splines to evaluate the assumption of a linear relationship between PFAS and antibody concentrations. Second, model sensitivities to additional potential confounding were assessed by including the following additional covariates in the regression models: BMI at age 28 years, serum PCB concentrations at birth and age 28 years, smoking status, and time interval between vaccination and blood sample collection. BMI has been associated with human immune function (Ilavská et al. 2012; Dobner and Kaser 2018), and has also been associated with PFAS exposures (Cardenas et al. 2018). Therefore, it was unclear whether BMI may act as a confounder or a mediator in the association between PFASs and immune function. Both PCB exposure and smoking have been associated with immune function. Further, since the variation of time interval between vaccination and blood sample collection may affect serum antibody concentrations, it was important to assess its potential role as a possible confounder. As an additional sensitivity analysis, the analytical sample was restricted to cohort members without diabetes due to the possible impacts on the immune system (Berbudi et al. 2020).

Lastly, a matched-sampling approach was utilized to evaluate whether the estimated associations were biased by potential covariate imbalance across PFAS exposure levels (Rubin 2008). This model was applied only to hepatitis type A and B analyses due to limited sample size in diphtheria and tetanus analysis. High and low PFAS exposures were defined at each timepoint based on the median of serum PFOA concentration, and logistic regression models were used to estimate the propensity score, i.e. the probability of having high PFOA exposure versus not given background covariates. A matched dataset was then created for each timepoint using propensity scores with a caliper width of 0.2 (Austin 2011), where the subjects with high and low PFOA exposure had similar covariate distributions. With an acceptable balance, the matched datasets could also be used for the analyses of other PFASs (i.e. PFOS, PFHxS, PFNA, PFDA). Finally, sex-adjusted linear regression models were used to evaluate continuous PFAS-antibody associations in these smaller matched datasets.

## Results

Table 1 shows major characteristics of 399 cohort members with hepatitis type A and B analyses and for 281 with diphtheria and tetanus data at age 28. At the follow-up examination, 3% (i.e. 11/399), 16% (62/399), 10% (27/281), and 0% (0/281) of the cohort members had antibody concentrations below the clinical protective levels for hepatitis type A, hepatitis type B, diphtheria, and tetanus, respectively. The average time intervals between vaccination and the follow-up blood sample collection were 124 days (range: 84–415 days) for hepatitis type A and B, and 191 days (range: 119–460 days) for diphtheria and tetanus. Follow-up serum concentrations of anti-HBs, anti-diphtheria, and anti-tetanus were higher among males, while females had higher serum anti-HAV. Table S1 shows the distributions of PFAS concentrations measured at five timepoints. Serum PFAS concentrations at ages 22 and 28 years were higher among males. Mean serum PFOS concentrations were the highest among the PFASs across all timepoints.

Figure S3 shows the within-visit and between-visit correlations of five PFASs among the cohort members included in hepatitis type A and B analyses. In general, within-visit correlations were higher for PFAS concentrations in adulthood (i.e. ages 22 and 28 years), with Spearman correlation coefficients from 0.4 (PFHxS-PFDA at age 22 years) to 0.9 (PFDA-PFNA at age 22 years). PFNA and PFDA were strongly correlated across all time points (Spearman rank-order correlation = 0.53–0.89). For associations between visits, concentrations at ages 22 and 28 years were the most highly-correlated. In contrast, cord-blood PFAS concentrations showed low correlations with PFAS concentrations at ages 7, 14, 22, and 28 years.

**Figure 2.**
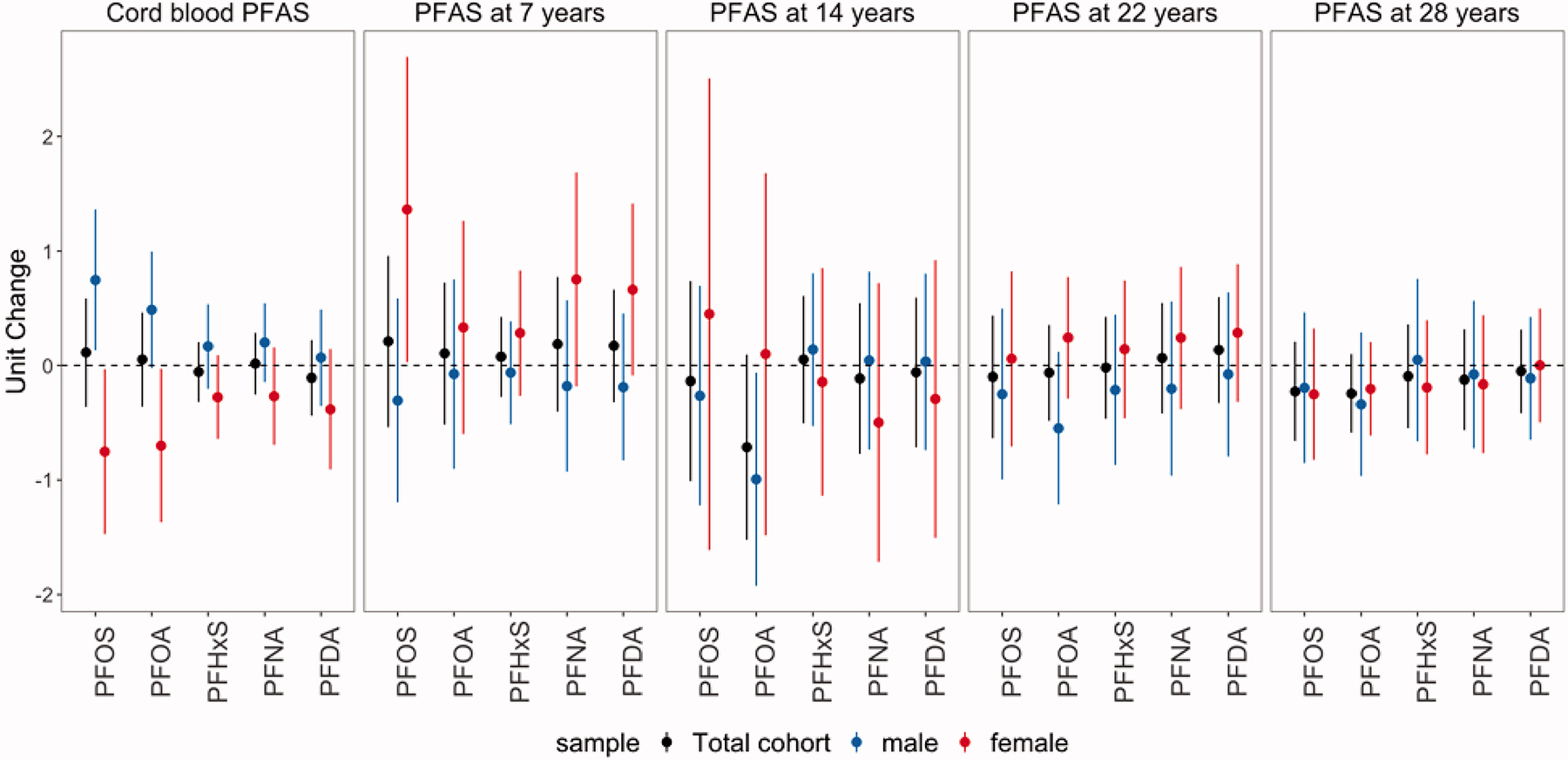
Unit change of serum anti-HAV concentrations (S/CO) at age 28 years per doubling of the cord-blood PFAS concentrations and serum PFAS concentrations at ages 7, 14, 22, and 28 years, overall and by sex.

**Figure 3.**
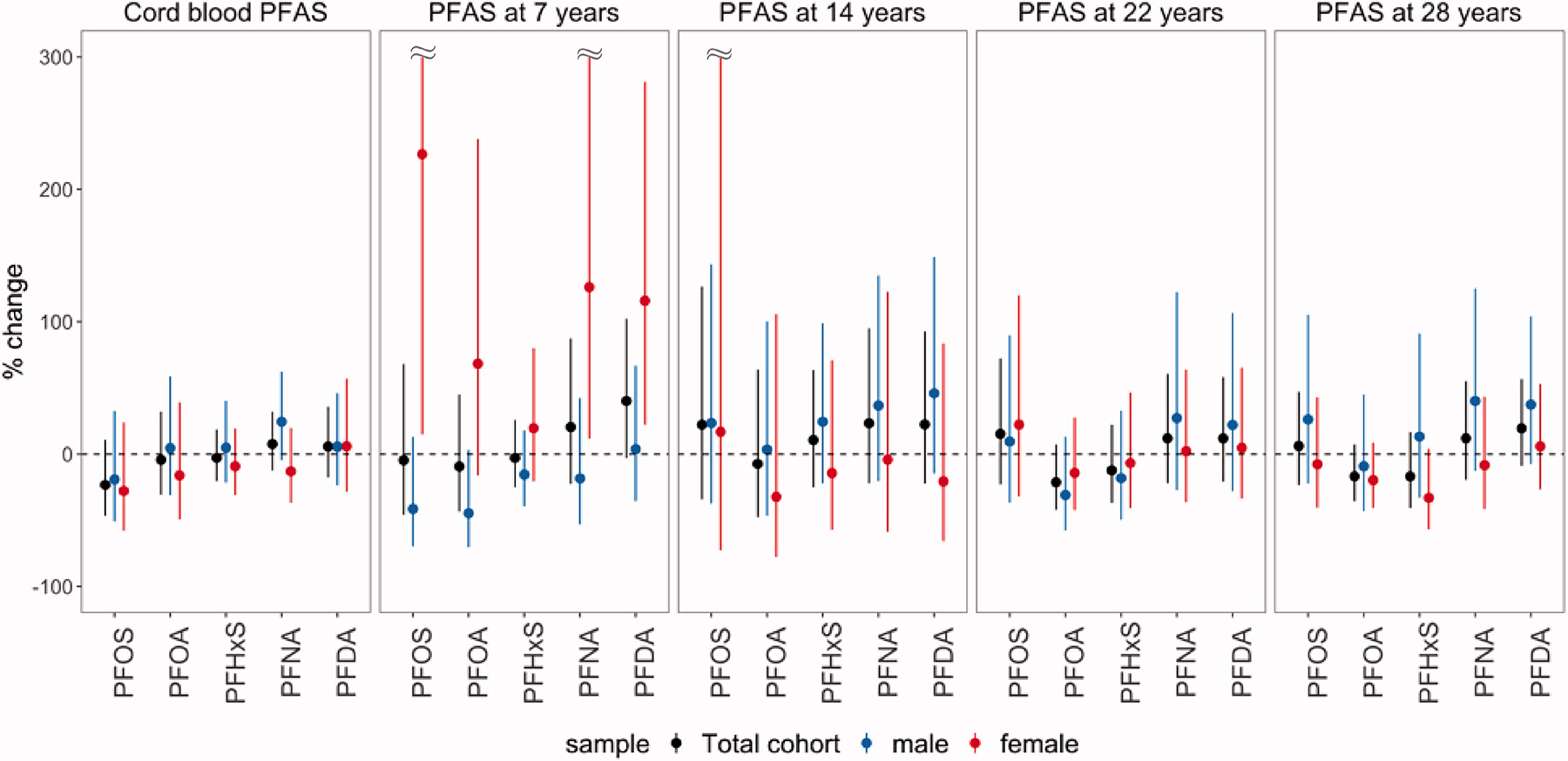
Percent change of serum anti-HBs concentrations at age 28 years per doubling of the cord-blood PFAS concentrations and serum PFAS concentrations at ages 7, 14, 22, and 28 years, overall and by sex.

The estimated associations of serum PFAS concentrations and serum antibody titers for hepatitis type A and B are summarized in Figures 2 and 3 and Tables S2 and S3. Overall, no evidence of associations based on a significance level threshold of 0.05 were found, but inverse trends were observed between anti-HAV and PFOA at ages 14 (S/CO change: −0.71; 95% CI: −1.52, 0.09) and 28 years (S/CO change: −0.24; 95% CI: −0.59, 0.10). Inverse trends were also observed between anti-HBs and PFOA at ages 22 (% change: −21.24; 95% CI: −42.20, 7.34) and 28 years (% change: −16.77; 95% CI: −35.47, 7.35).

The estimated associations between anti-HAV and anti-HBs and PFAS at birth, 7 and 14 years were modified by sex. While negative associations of anti-HAV with cord-blood PFOS and PFOA were estimated among women, positive associations among males (*P*_*interaction*_ for PFOS = 0.006; *P*_*interaction*_ for PFOA = 0.010) were noted. Additionally, positive associations of PFAS at age 7 years with anti-HAV and anti-HBs concentrations were estimated among females only. Further, a negative association between anti-HAV and PFOA at age 14 years was only observed among males, though the interaction between PFOA and sex was not significant (*P*_*interaction*_=0.24).

Estimated associations of PFAS exposure and serum concentrations of antibody against diphtheria and tetanus are summarized in Figure S4 and Tables S4 and S5. Positive associations of anti-diphtheria with cord-blood PFAS and PFAS were estimated at ages 22 and 28 years, with stronger associations with cord-blood PFAS for males. No evidence of associations were observed in relation to anti-tetanus.

In the GAMs with penalized thin-plate splines, 76% were fit with degrees of freedom less than two, and only one model suggested a non-linear association between PFNA at age 14 years and anti-HBs concentrations (Figure S5). In sensitivity analyses where BMI, smoking status, and time interval were included as covariates in separate regression models, the association estimates of PFASs on antibody concentrations were of a similar magnitude and in the same direction, thus suggesting no important confounding by these variables (data not shown). However, serum-PCB concentrations at birth and age 28 years seemed to confound effects of some PFAS exposures (Figures S6–S7). For example, after additionally adjusting for serum PCB at birth or age 28 years, the associations of PFNA and PFDA at age 7 years with anti-HBs were attenuated, while the associations were stronger on anti-diphtheria and anti-tetanus after. Additionally, in sensitivity analyses excluding three cohort members with diabetes, similar results with consistent directionality and significance were observed (data not shown).

In the Rubin Causal analysis using the matched datasets, distributions of covariates were balanced between high and low PFOA exposure (Figures S8–S12). Table S6 shows the sample sizes of the matched datasets at each timepoint. Overall, results from this analysis were generally similar to early primary results, suggesting estimated associations from the main analysis were not likely to be biased by covariance imbalance (Figures S13–S15). While association estimates of PFASs at ages 7 and 14 years especially among females were relatively different, this could have possibly been due to small sample sizes in the matched datasets.

## Discussion

In this study of adults from a prospective birth cohort in the Faroe Islands, associations of cord-blood PFAS concentrations and serum PFAS concentrations at ages 7, 14, 22, and 28 years were estimated with vaccine antibody concentrations at age 28 years. Overall, limited evidence were found regarding inverse associations between exposure to PFASs and antibody concentrations based on a significance level threshold of 0.05, although borderline inverse trends were observed between PFOA at ages 14 and 28 years and anti-HAV as well as between PFOA at ages 22 and 28 years and anti-BHs. Furthermore, sex-specific associations were identified for prenatal and early-life exposure to PFASs. For example, negative associations of anti-HAV with cord-blood PFOS and PFOA were observed among females, while associations appeared to be positive among males.

A major advantage of the current study was the use of antibody response to immunization as a clinically-relevant marker to reflect immune system function (DeWitt et al. 2019). A vaccine response is a well-defined stimulation of the adaptive immune system; evaluations of such responses were recommended at an international symposium on Principles and Methods for Assessing Immunotoxicity held in Bilthoven – as well as by the World Health Organization (van Loveren et al. 1999). Another strength of this study is that the subjects received the same dose of the same antigens at the same age. Specifically, subjects included in the hepatitis type A and B analyses had not received previous vaccination against hepatitis type A or B (thus representing a *de novo* antibody response). However, it is possible some subjects may have been vaccinated against diphtheria and tetanus during emergency room visits for cuts and other injuries (though the present study contained criteria to exclude participants with prior booster vaccinations against tetanus and diphtheria). Despite the inclusion criteria applied, recent boosters may have contributed to some unexpected positive associations between PFAS exposure and fairly low anti-diphtheria titers. Of note, a previous study of tetanus and diphtheria antibody concentrations in teenagers showed scattered associations with PFAS exposure profiles only when related to previous antibody data (Grandjean et al. 2017a).

In the current study, serum antibody concentrations against hepatitis type A and B appeared to be negatively affected by recent exposures to PFOA (i.e. at ages 14, 22, and 28 years). The findings here are supported by a large population survey of adults (aged 18 years or older) living in Ohio or West Virginia conducted by the C8 Science Panel; that study found that elevated serum PFOA concentrations were associated with reduced antibody response to the influenza A H3N2 virus vaccination with an inactivated intramuscular trivalent vaccine, although another virus strain did not show this association (Looker et al. 2014). A more recent study of 1,193 adults from the U.S. National Health and Nutrition Examination Survey similarly reported an inverse association between serum PFOA and rubella antibody concentrations among males (Pilkerton et al. 2018). In a small cross-sectional study of 12 healthy adults in Denmark, significant inverse associations were observed between six different PFASs and the vaccine-induced increases in diphtheria and tetanus titers, and the associations were stronger for the longer-chain PFASs (Kielsen et al. 2016). Further, a recent study of 605 older adults in the Isomer of C8 Health Project in China found that exposure to five PFASs was associated with lower hepatitis B surface antibody levels, although no association was observed in relation to PFOA. In contrast, in a small study of 78 healthy U.S. adults (aged 18–49 years), no significant association was found between serum PFAS concentrations and antibody response to influenza A H1N1 virus after FluMist vaccination (Stein et al. 2016). The somewhat inconsistent findings across studies may be due to different PFAS exposure profiles, antibody types, time intervals since last vaccination, and exposure windows assessed.

Potential biological mechanisms underlying associations between PFAS exposures and immune system functions remain unclear. Some studies suggested that important pathways may involve anti-inflammatory effects that evolve, in part, through activation of peroxisome proliferator-activated receptor (PPAR)-α and cytokine/chemokine suppression regulated by activation of nuclear factor (NF)-κB (Corsini et al. 2014; DeWitt et al. 2016, 2019; Pennings et al. 2016). The current analyses of sex-specific effects of PFAS exposures also suggested that associations between prenatal and early life exposures to PFASs on vaccine responses differed by sex. A recent review suggested that epigenetic alteration and activation of estrogen receptor are possible mechanisms underlying sex-specific associations of environmental chemicals with the outcome of immune responses (Edwards et al. 2018). Further, in a study of 1,000 healthy adults in Helsinki, expression of immune-related genes induced by viral stimulation was shown to vary by sex (Piasecka et al. 2018).

As with any study, there are important limitations to consider here. First, an antibody concentration measured at a particular point in time does not represent the complete trajectory of antibody response and long-term protection against a specific disease. However, this is con-sidered to have minimal impact on the present results since antibody titration curves typically show a plateau after a certain concentration is reached (Kielsen et al. 2016). Second, PFAS concentrations measured from cord-blood may not reflect prenatal exposure during the whole course of pregnancy. Additionally, the current study results may be subject to multiple testing considerations that could lead to a Type I error. Still, it is unlikely that the significant immunotoxic effects of PFOA exposures here were observed by chance, given the consistency in associations across three time points (i.e. 14, 22, and 28years). Lastly, residual confounding due to unmeasured variables. like socioeconomic status-related variables, may remain in this study.

The present study longitudinally-investigated potential immunotoxic effects of PFASs in adults as assessed via any changes in PFAS-associated deficient vaccine responses. Overall, limited evidence was shown for associations between PFAS exposures and decreased antibody concentrations. Potential inverse trends of recent PFAS exposure with lower *de novo* vaccine responses to hepatitis type A and B at ages 28 years were noted; these mandate further investigation. Lastly, sex-specific associations with vaccine responses were observed for prenatal and early-life exposures to PFASs. Future prospective studies are warranted to confirm these findings and further investigate potential sex-specific effects of PFASs, vaccine response trajectories, and immune-related health outcomes in adults.

## Supplementary Material

Supplementary Material

## Figures and Tables

**Table 1.** Characteristics of participants included in analysis of hepatitis type A and B, diphtheria, and tetanus with PFAS concentrations at age 28years.

Characteristics	Hepatitis type A and B analyses				Diphtheria and tetanus analyses			
	Total cohort (*n* = 399)	Male (*n* = 220, 55.1%)	Female (*n* = 179; 44.9%)	p-Value^a^	Total cohort (n = 281 )	Male (*n* = 133, 47.3%)	Female (n = 148, 52.7%)	p-Value^a^

BMI, n (%) (kg/m^2^)				0.47				0.05
<18	1 (0.3)	0 (0.0)	1 (0.6)		2 (0.7)	0 (0.0)	2 (1.4)	
18–25	182 (45.6)	98 (44.5)	84 (46.9)		135 (48.0)	56 (42.1)	79 (53.4)	
≥25	216 (54.1)	122 (55.5)	94 (52.5)		144 (51.2)	77 (57.9)	67 (45.3)	
Smoking status, *n* (%)				0.14				0.38
Current	206 (51.6)	105 (47.7)	101 (56.4)		140 (49.8)	72 (54.1)	68 (45.9)	
Ever	52 (13.0)	29 (13.2)	23 (12.8)		37 (13.2)	15 (11.3)	22 (14.9)	
Never	139 (34.8)	86 (39.1)	53 (29.6)		103 (36.7)	46 (34.6)	57 (38.5)	
Missing	2 (0.5)	0 (0.0)	2 (1.1)		1 (0.4)	0 (0.0)	1 (0.7)	
Antibody concentrations, median (IQR)								
Baseline anti-diphtheria (IU/ mL)	-	-	-		0.01 (0.05)	0.01 (0.05)	0.01 (0.05)	0.23
Follow-up anti-diphtheria (IU/ mL)	-	-	-		1.10 (2.92)	1.60 (3.93)	0.57 (2.10)	<0.001
Baseline anti-tetanus (IU/ mL)	-	-	-		0.12 (0.52)	0.12 (0.55)	0.13 (0.52)	0.61
Follow-up anti-tetanus (IU/ mL)	-	-	-		6.60 (12.80)	7.50 (13.8)	5.65 (8.10)	0.03
Follow-up anti-FIAV (S/CO)	6.11 (4.00)	5.61 (3.78)	6.85 (3.73)	0.002	-	-	-	
Follow-up anti-FIBs (mlU/mL)	56.5 (143.60)	66.76 (159.48)	44.28 (113.96)	0.006	-	-	-	

a Chi-square test for BMI and smoking status and Wilcoxon rank-sum test for antibody and PFAS concentrations.

b IQR: interquartile range; S/CO: signal-to-cutoff ratio; PFAS: per- and polyfluoroalkyl substance; PFOS: perfluorooctane sulfonate; PFOA: perflourooctanoate; PFHxS: perfluorohexanesulfonic acid; PFNA: perfluoronona-noate; PFDA: perfluorodecanoate.
