# Supplementary material for: Serum vaccine antibody concentrations in adults exposed to per- and polyfluoroalkyl substances: A birth cohort in the Faroe Islands
Source: J Immunotoxicol. Author manuscript; Available in PMC 2023 May 23. (PMC10204592; doi:10.1080/1547691X.2021.1922957)
Supplement: Supplementary Material [file NIHMS1894146-supplement-Supplementary_Material.docx]

**Supplemental Materials**

**Serum vaccine antibody concentrations in adults exposed to per- and polyfluoroalkyl substances: a birth cohort in the Faroe Islands**

Yu-Hsuan Shih,^1*^ Annelise J. Blomberg,^1^ Marie-Abèle Bind,^2^ Dorte Holm,^3^ Flemming Nielsen,^6^ Carsten Heilmann,^4^ Pal Weihe,^5,7^ Philippe Grandjean^1,6^

^1^ Department of Environmental Health, Harvard T.H. Chan School of Public Health, Boston, MA, United States

^2^ Department of Medicine, Massachusetts General Hospital and Harvard Medical School, Boston, MA, United States

^3^ Department of Clinical Immunology, Odense University Hospital, Odense, Denmark

^4^ National University Hospital, Copenhagen, Denmark

^5^ University of the Faroe Islands, Torshavn, Faroe Islands

^6^ Department of Environmental Medicine, University of Southern Denmark, Odense, Denmark

^7^ Department of Occupational Medicine and Public Health, The Faroese Hospital System, Torshavn, Faroe Islands

**Supplemental Figures**

Figure S1. Flow chart of sample selection for hepatitis type A and B analysis …………….. 3

Figure S2. Flow chart of sample selection for diphtheria and tetanus analysis ……………... 4

Figure S3. Spearman correlation coefficients between PFAS concentration. Analytical sample for hepatitis type A and B analysis was used ………………………………………………... 5

Figure S4. Percent change of the follow-up serum anti-diphtheria and anti-tetanus concentrations at age 28 years per doubling of the cord blood PFAS concentrations and serum PFAS concentrations at ages 7, 14, 22, and 28 years ………………………………………... 6

Figure S5. Non-linear association between PFNA at age 14 years and anti-HBs concentrations based on penalized thin-plate splines ………………………………………………………... 7

Figure S6. Comparison of effect estimates from sex-adjusted models and models additionally including polychlorinated biphenyls (PCB) concentrations at birth and age 28 years for the associations between PFAS exposure and follow-up antibody concentrations of hepatitis type A and B ………………………………………………………………………………………. 8

Figure S7. Comparison of effect estimates from sex-adjusted models and models additionally including polychlorinated biphenyls (PCB) concentrations at birth and age 28 years for the associations between PFAS exposure and follow-up antibody concentrations of diphtheria and tetanus ………………………………………………………………………………………... 9

Figure S8. Love plot of the absolute standardized difference in covariate means between the high PFOA exposed and low PFOA exposed at birth, before and after matching on propensity scores ………………………………………………………………………………………... 10

Figure S9. Love plot of the absolute standardized difference in covariate means between the high PFOA exposed and low PFOA exposed at age 7 years, before and after matching on propensity scores ………………………………………………………………………….… 11

Figure S10. Love plot of the absolute standardized difference in covariate means between the high PFOA exposed and low PFOA exposed at age 14 years, before and after matching on propensity scores ……………………………………………………………………………. 12

Figure S11. Love plot of the absolute standardized difference in covariate means between the high PFOA exposed and low PFOA exposed at age 22 years, before and after matching on propensity scores ……………………………………………………………………………. 13

Figure S12. Love plot of the absolute standardized difference in covariate means between the high PFOA exposed and low PFOA exposed at age 28 years, before and after matching on propensity scores ……………………………………………………………………………. 14

Figure S13. Unit change of serum anti-HAV and percent change of serum anti-HBs at age 28 years per doubling of the cord blood PFAS concentrations and serum PFAS concentrations at ages 7, 14, 22, and 28 years in the overall study population. Results are shown for our primary models and causal models (propensity score-matched dataset) …………………………….. 15

Figure S14. Unit change of serum anti-HAV and percent change of serum anti-HBs at age 28 years per doubling of the cord blood PFAS concentrations and serum PFAS concentrations at ages 7, 14, 22, and 28 years in males. Results are shown for our primary models and causal models (propensity score-matched dataset) ………………………………………………… 16

Figure S15. Unit change of serum anti-HAV and percent change of serum anti-HBs at age 28 years per doubling of the cord blood PFAS concentrations and serum PFAS concentrations at ages 7, 14, 22, and 28 years in females. Results are shown for our primary models and causal models (propensity score-matched dataset) ………………………………………………… 17

**Supplemental Tables**

Table S1. Median and interquartile range (IQR) of PFAS concentrations at ages 7, 14, and 22 years, overall and by sex ……………………………………………………………………. 18

Table S2. Unit change of serum anti-HAV concentrations (S/CO) at age 28 years per doubling of the cord blood PFAS concentrations and serum PFAS concentrations at ages 7, 14, 22, and 28 years ……………………………………………………………………………………... 19

Table S3. Percent change of serum anti-HBs concentrations at age 28 years per doubling of the cord blood PFAS concentrations and serum PFAS concentrations at ages 7, 14, 22, and 28 years ………………………………………………………………………………………… 20

Table S4. Percent change of the follow-up serum anti-diphtheria concentrations at age 28 years per doubling of the cord blood PFAS concentrations and serum PFAS concentrations at ages 7, 14, 22, and 28 years ………………………………………………………………… 21

Table S5. Percent change of the follow-up serum anti-tetanus concentrations at age 28 years per doubling of the cord blood PFAS concentrations and serum PFAS concentrations at ages 7, 14, 22, and 28 years …………………………………………………………………....... 22

Table S6. Sample size of the matched datasets for the Rubin Causal analysis ……………... 23

Did not receive hepatitis type A or B vaccine before (N=401)

Without missing data on antibody concentrations (N=400)

Hepatitis Type A final sample:

- Cord blood PFASs (N=388)

- PFASs at age 7 years (N=280)

- PFASs at age 14 years (N=177)

- PFASs at age 22 years (N=361)

- PFASs at age 28 years (N=399)

Hepatitis Type B final sample:

- Cord blood PFASs (N=375)

- PFASs at age 7 years (N=269)

- PFASs at age 14 years (N=172)

- PFASs at age 22 years (N=350)

- PFASs at age 28 years (N=386)

Exclude participants with extreme anti-HBs concentrations (n=13)

Cohort members who had completed vaccination for hepatitis type A and B at age 28 years (N=454)

Figure S1. Flow chart of sample selection for hepatitis type A and B analysis

Cohort members vaccinated for diphtheria and tetanus at age 28 years (N=593)

Without missing data on diphtheria and tetanus antibody levels at age 28 years (N=518)

Antibody concentrations of diphtheria or tetanus at age 7 years ≥ the baseline antibody levels at age 28 years (N=118)

Baseline antibody concentrations of diphtheria < 0.5 IU/mL or tetanus < 2 IU/mL at age 28 years (N=165)

Without antibody concentrations at age 7 years (N=263)

With antibody concentrations at age 7 years (N=255)

Baseline antibody levels of diphtheria or tetanus lower than the follow-up antibody levels (N=281)

Final sample:

- PFASs at age 0 years (N=276)

- PFASs at age 7 years (N=173)

- PFASs at age 14 years (N=127)

- PFASs at age 22 years (N=250)

- PFASs at age 28 years (N=281)

Figure S2. Flow chart of sample selection for diphtheria and tetanus analysis


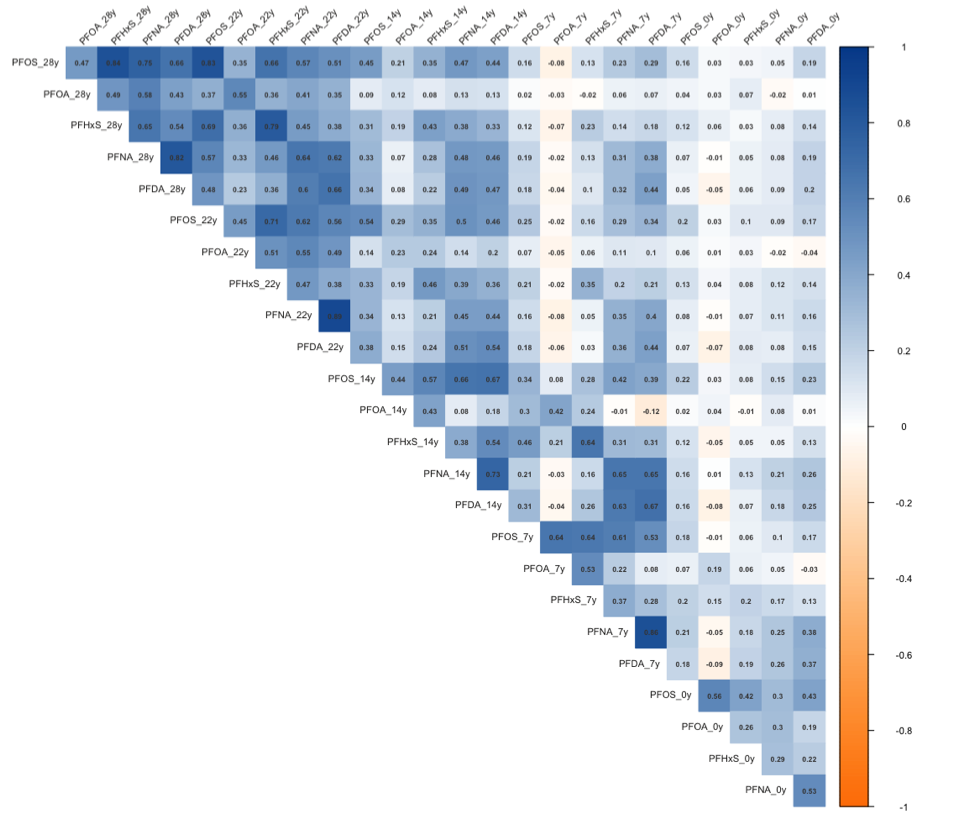


Figure S3. Spearman correlation coefficients between PFAS concentration. Analytical sample for hepatitis type A and B analysis was used


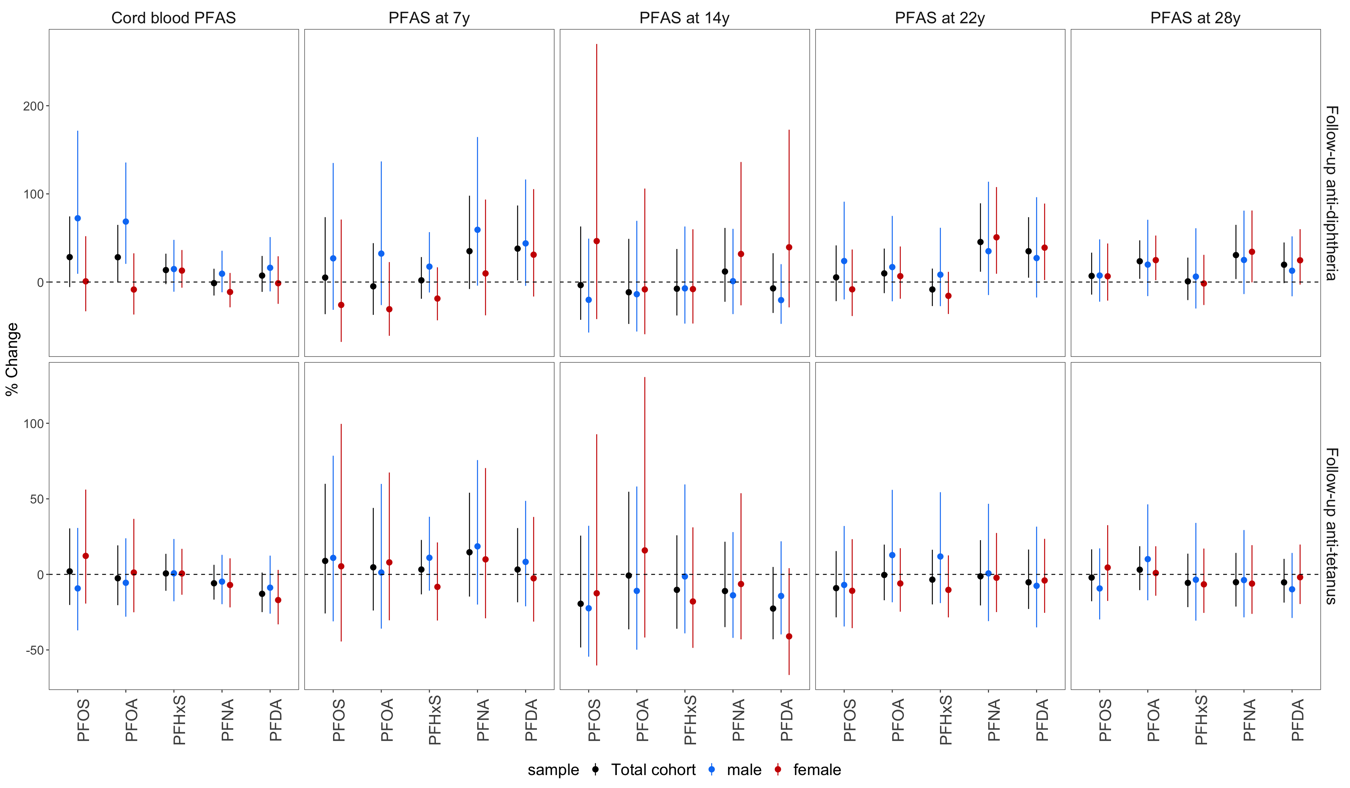


Figure S4. Percent change of the follow-up serum anti-diphtheria and anti-tetanus concentrations at age 28 years per doubling of the cord blood PFAS concentrations and serum PFAS concentrations at ages 7, 14, 22, and 28 years


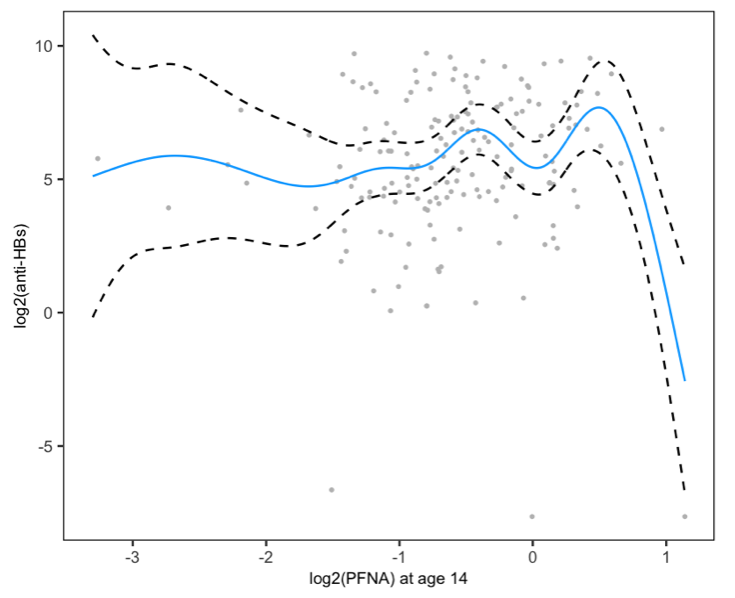


Figure S5. Non-linear association between PFNA at age 14 years and anti-HBs concentrations based on penalized thin-plate splines


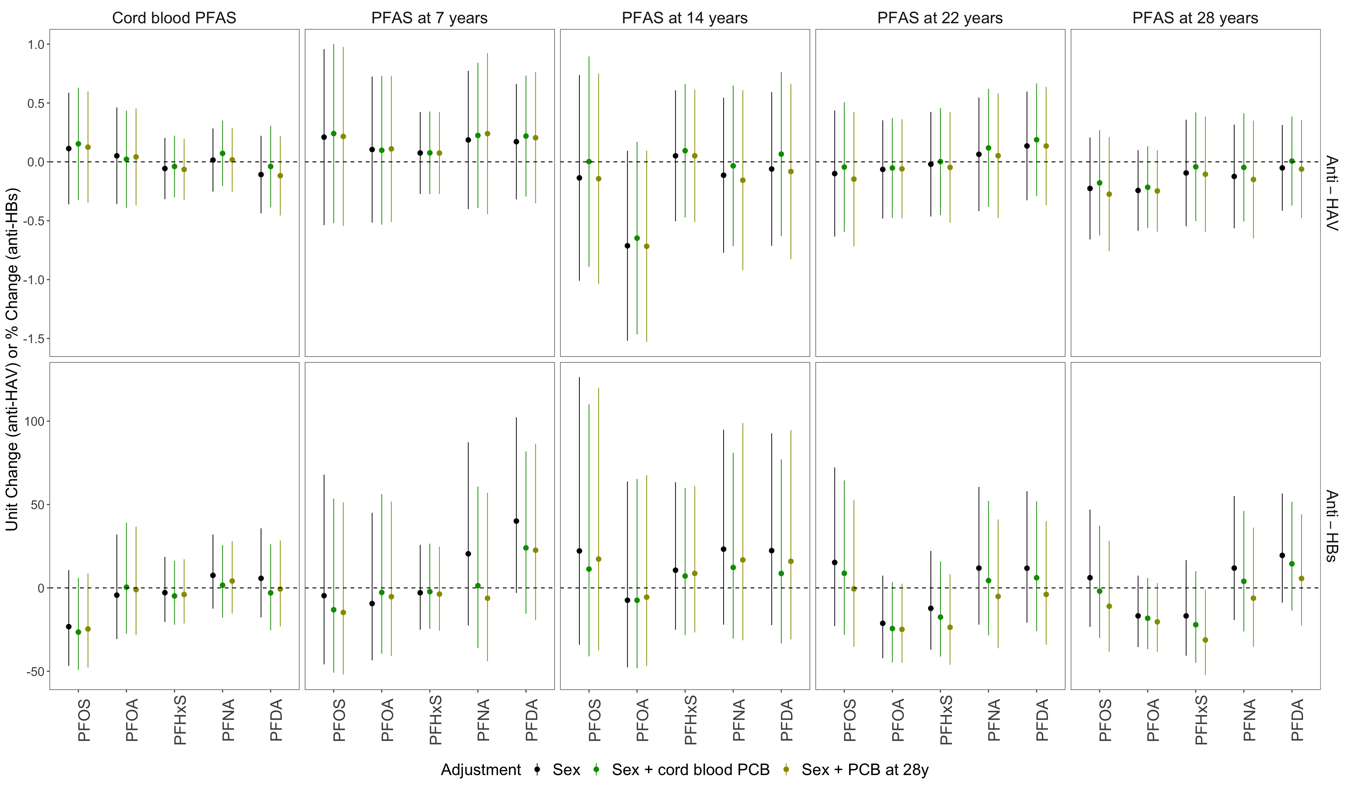


Figure S6. Comparison of effect estimates from sex-adjusted models and models additionally including polychlorinated biphenyls (PCB) concentrations at birth and age 28 years for the associations between PFAS exposure and follow-up antibody concentrations of hepatitis type A and B


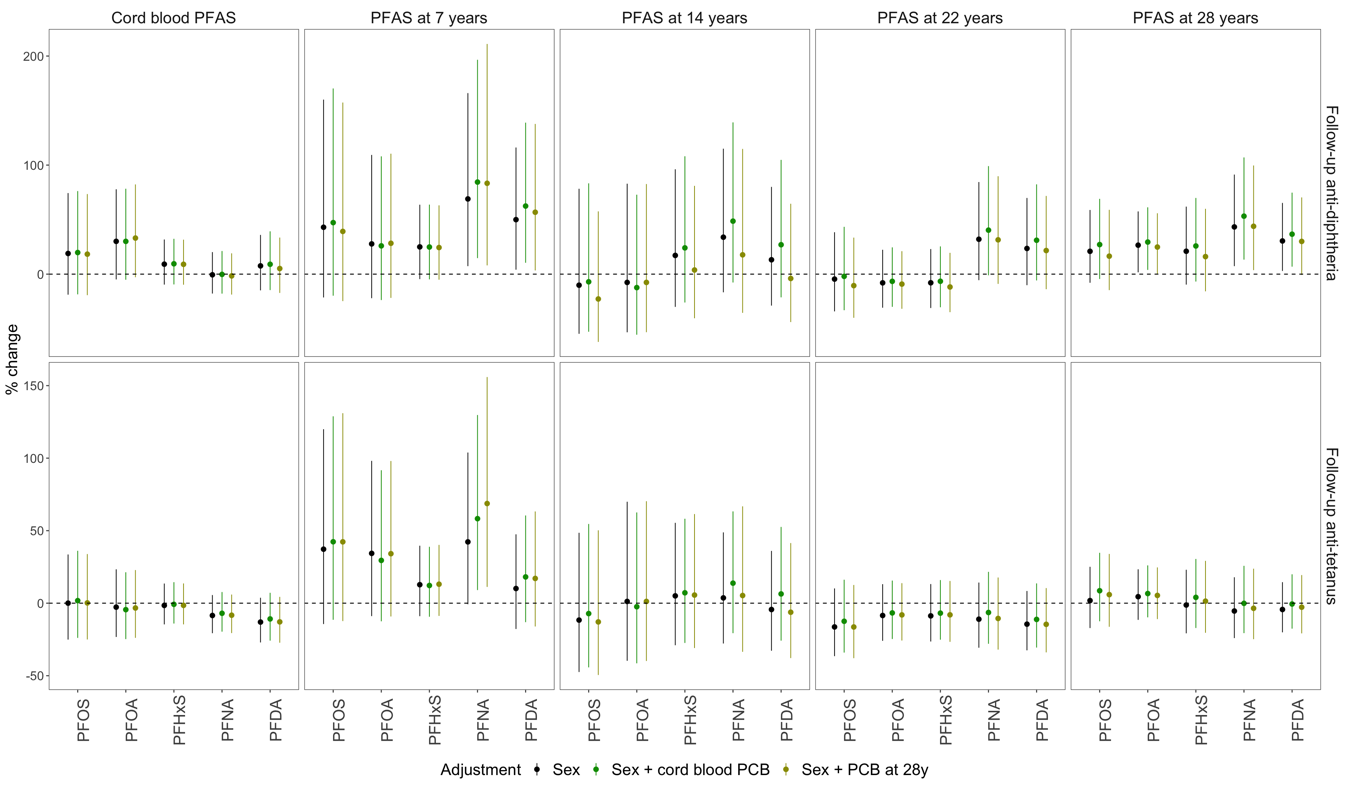


Figure S7. Comparison of effect estimates from sex-adjusted models and models additionally including polychlorinated biphenyls (PCB) concentrations at birth and age 28 years for the associations between PFAS exposure and follow-up antibody concentrations of diphtheria and tetanus


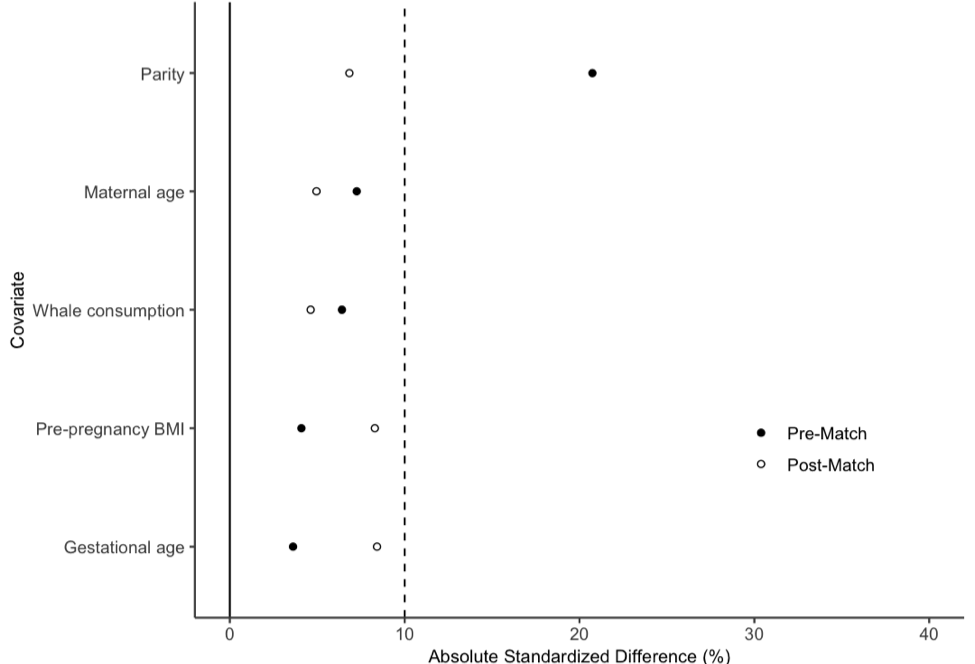


Figure S8. Love plot of the absolute standardized difference in covariate means between the high PFOA exposed and low PFOA exposed at birth, before and after matching on propensity scores.


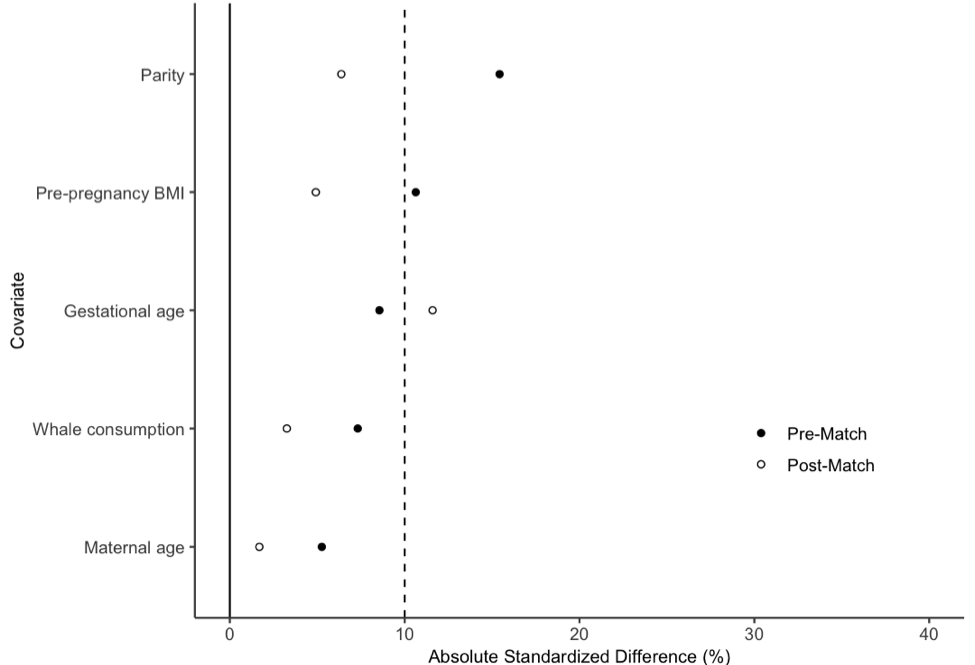


Figure S9. Love plot of the absolute standardized difference in covariate means between the high PFOA exposed and low PFOA exposed at age 7 years, before and after matching on propensity scores.


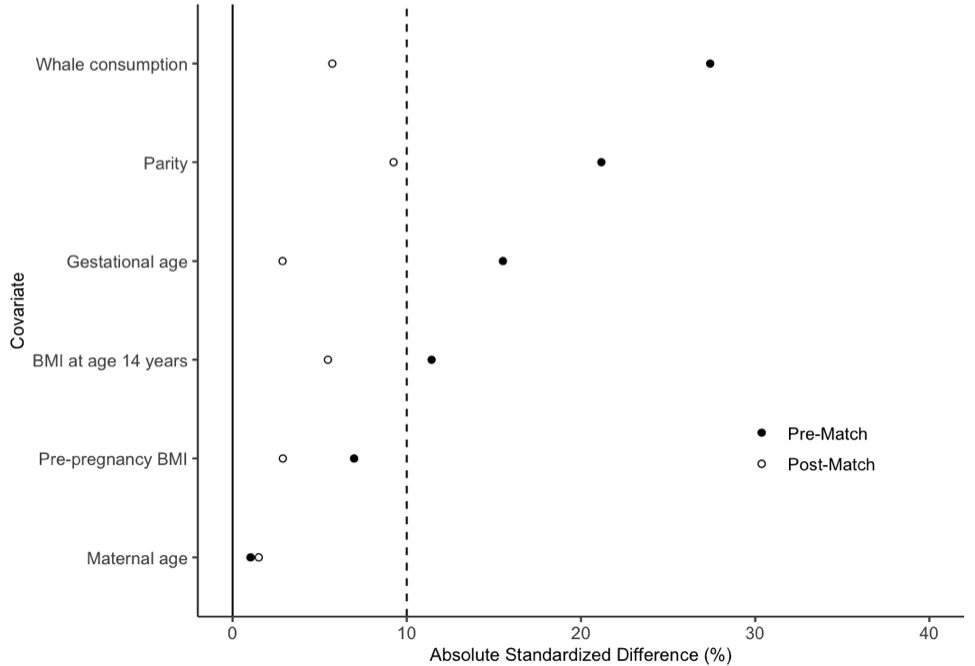


Figure S10. Love plot of the absolute standardized difference in covariate means between the high PFOA exposed and low PFOA exposed at age 14 years, before and after matching on propensity scores.


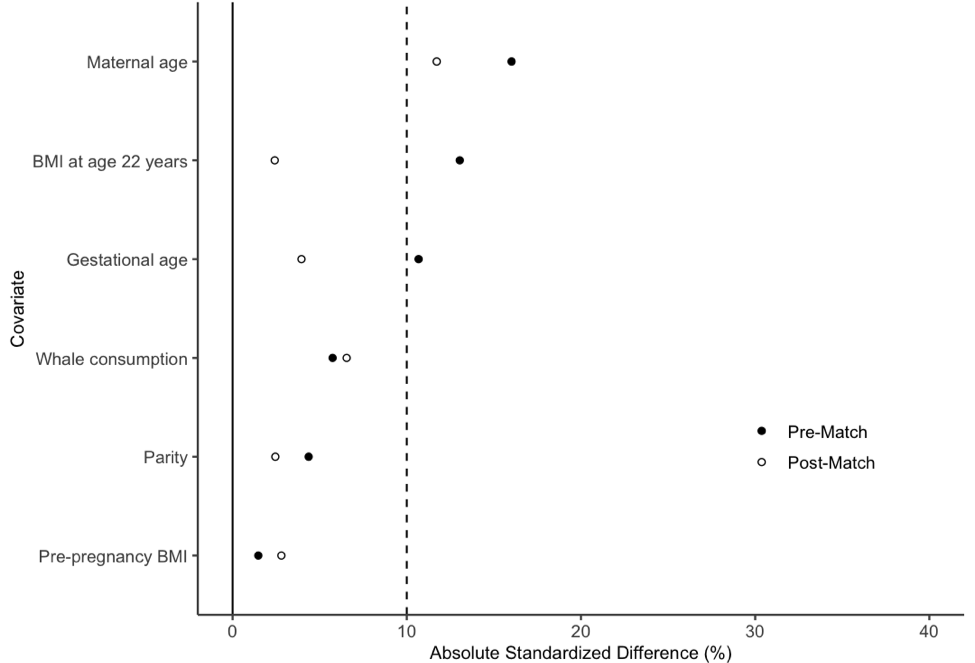


Figure S11. Love plot of the absolute standardized difference in covariate means between the high PFOA exposed and low PFOA exposed at age 22 years, before and after matching on propensity scores.


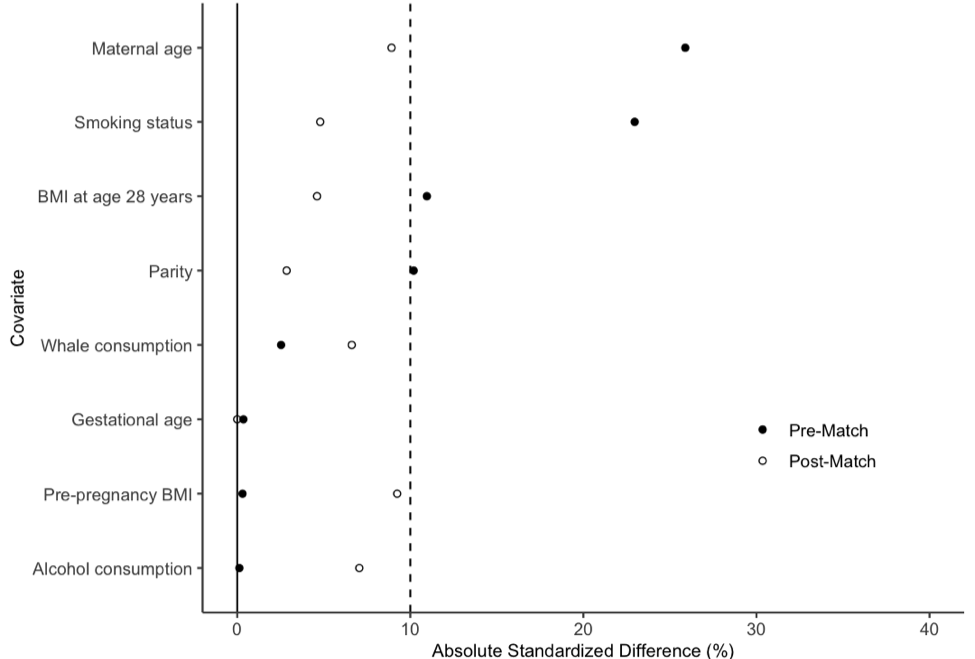


Figure S12. Love plot of the absolute standardized difference in covariate means between the high PFOA exposed and low PFOA exposed at age 28 years, before and after matching on propensity scores.


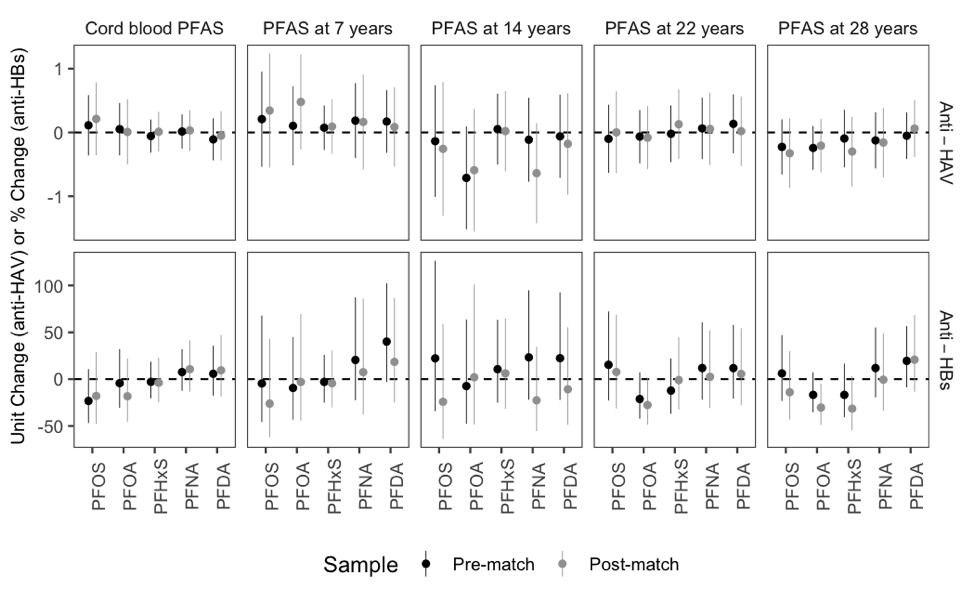


Figure S13. Unit change of serum anti-HAV and percent change of serum anti-HBs at age 28 years per doubling of the cord blood PFAS concentrations and serum PFAS concentrations at ages 7, 14, 22, and 28 years in the overall study population. Results are shown for our primary models and causal models (propensity score-matched dataset)


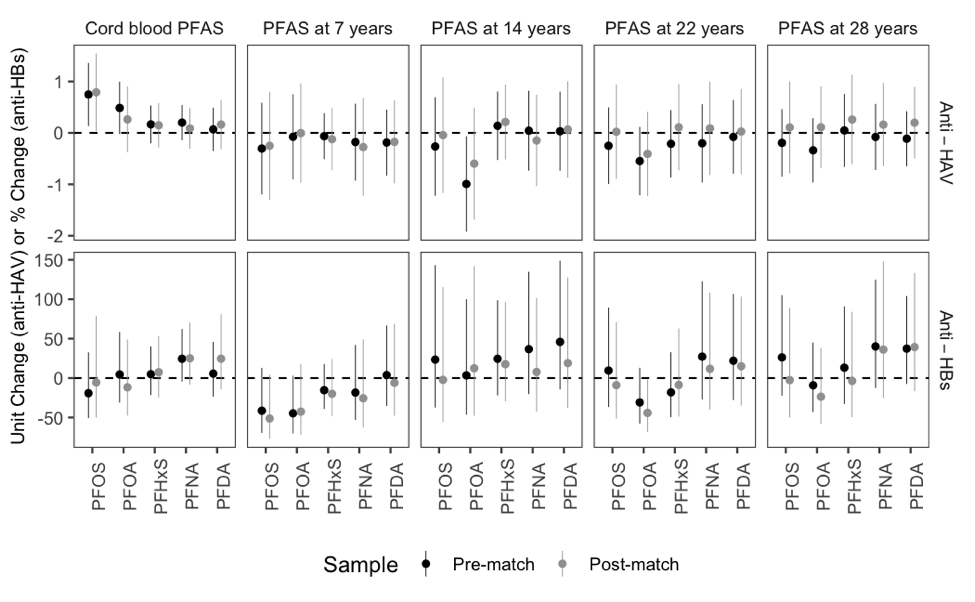


Figure S14. Unit change of serum anti-HAV and percent change of serum anti-HBs at age 28 years per doubling of the cord blood PFAS concentrations and serum PFAS concentrations at ages 7, 14, 22, and 28 years in males. Results are shown for our primary models and causal models (propensity score-matched dataset)


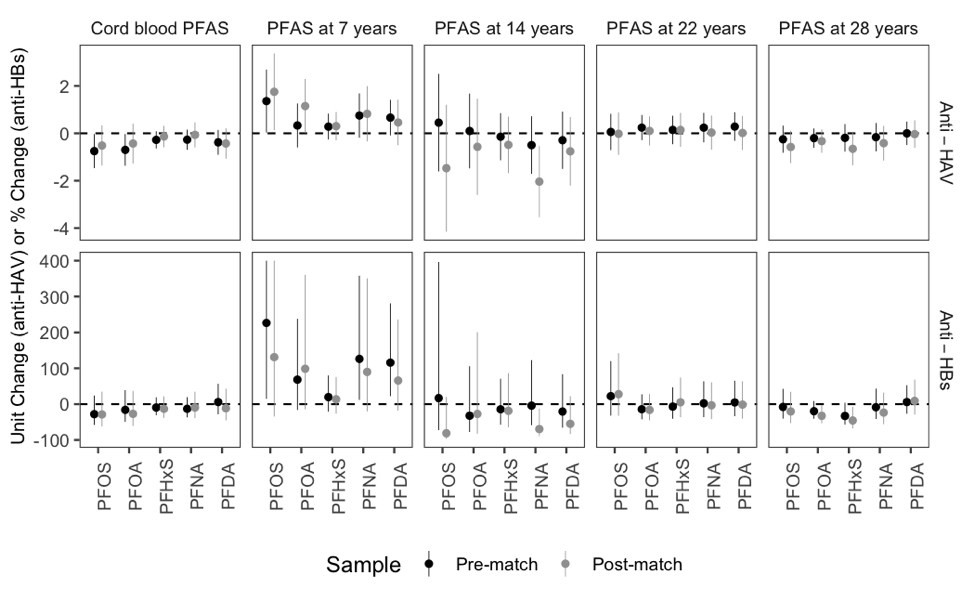


~

~

Figure S15. Unit change of serum anti-HAV and percent change of serum anti-HBs at age 28 years per doubling of the cord blood PFAS concentrations and serum PFAS concentrations at ages 7, 14, 22, and 28 years in females. Results are shown for our primary models and causal models (propensity score-matched dataset).

| Table S1. Median and interquartile range (IQR) of PFAS concentrations at ages 7, 14, and 22 years, overall and by sex. | | | | | | | | | |  |
| --- | --- | --- | --- | --- | --- | --- | --- | --- | --- | --- |
| PFAS (ng/mL) | Hepatitis type A and B analysis^a^ | | | |  | Diphtheria and tetanus analysis^b^ | | | |  |
|  | Total cohort | Male | Female | P-value^c^ |  | Total cohort | Male | Female | P-value^c^ |  |
| PFOS at birth | 5.96 (3.09) | 5.91 (2.95) | 6.20 (3.15) | 0.87 |  | 5.97 (2.83) | 5.95 (2.91) | 6.09 (2.87) | 0.85 |  |
| PFOS at 7 y | 31.89 (13.37) | 31.48 (13.39) | 32.09 (12.32) | 0.18 |  | 31.25 (12.41) | 30.51 (12.07) | 31.94 (13.75) | 0.16 |  |
| PFOS at 14 y | 31.29 (9.62) | 31.99 (12.21) | 30.56 (6.75) | 0.14 |  | 30.64 (9.85) | 32.09 (12.50) | 29.46 (7.18) | 0.05 |  |
| PFOS at 22 y | 12.55 (7.24) | 14.86 (6.73) | 9.94 (5.44) | <0.001 |  | 12.14 (7.60) | 15.12 (7.28) | 9.95 (5.83) | <0.001 |  |
| PFOS at 28 y | 6.85 (5.29) | 9.14 (4.86) | 4.59 (3.49) | <0.001 |  | 6.10 (5.49) | 9.31 (5.18) | 4.44 (2.93) | <0.001 |  |
| PFOA at birth | 1.11 (0.62) | 1.16 (0.64) | 1.06 (0.60) | 0.12 |  | 1.08 (0.58) | 1.15 (0.63) | 1.04 (0.50) | 0.07 |  |
| PFOA at 7 y | 5.11 (2.45) | 5.07 (2.58) | 5.31 (2.29) | 0.49 |  | 4.91 (1.95) | 4.88 (1.94) | 5.31 (2.13) | 0.76 |  |
| PFOA at 14 y | 4.98 (2.11) | 5.23 (2.11) | 4.59 (1.74) | 0.06 |  | 5.04 (2.05) | 5.22 (2.58) | 4.62 (1.71) | 0.28 |  |
| PFOA at 22 y | 2.96 (1.69) | 3.13 (1.59) | 2.66 (1.54) | <0.001 |  | 2.96 (1.70) | 3.13 (1.47) | 2.67 (1.92) | 0.02 |  |
| PFOA at 28 y | 1.28 (0.90) | 1.44 (0.75) | 1.06 (0.89) | <0.001 |  | 1.24 (0.88) | 1.37 (0.62) | 1.07 (0.98) | <0.001 |  |
| PFHxS at birth | 0.21 (0.14) | 0.20 (0.13) | 0.23 (0.17) | 0.02 |  | 0.20 (0.15) | 0.19 (0.13) | 0.21 (0.17) | 0.06 |  |
| PFHxS at 7 y | 0.90 (0.42) | 0.91 (0.47) | 0.89 (0.41) | 0.82 |  | 0.90 (0.41) | 0.92 (0.42) | 0.90 (0.37) | 0.38 |  |
| PFHxS at 14 y | 0.63 (0.34) | 0.70 (0.37) | 0.59 (0.27) | 0.08 |  | 0.59 (0.36) | 0.67 (0.37) | 0.55 (0.28) | 0.04 |  |
| PFHxS at 22 y | 0.54 (0.37) | 0.66 (0.36) | 0.40 (0.25) | <0.001 |  | 0.52 (0.38) | 0.66 (0.35) | 0.39 (0.25) | <0.001 |  |
| PFHxS at 28 y | 0.41 (0.30) | 0.54 (0.24) | 0.25 (0.16) | <0.001 |  | 0.37 (0.33) | 0.56 (0.26) | 0.23 (0.15) | <0.001 |  |
| PFNA at birth | 0.11 (0.11) | 0.10 (0.11) | 0.11 (0.12) | 0.72 |  | 0.10 (0.12) | 0.11 (0.11) | 0.10 (0.12) | 0.57 |  |
| PFNA at 7 y | 0.68 (0.39) | 0.67 (0.38) | 0.69 (0.41) | 0.08 |  | 0.67 (0.39) | 0.60 (0.40) | 0.73 (0.44) | 0.02 |  |
| PFNA at 14 y | 0.66 (0.37) | 0.69 (0.46) | 0.62 (0.22) | 0.14 |  | 0.66 (0.35) | 0.69 (0.47) | 0.64 (0.19) | 0.27 |  |
| PFNA at 22 y | 0.94 (0.50) | 1.03 (0.50) | 0.83 (0.52) | <0.001 |  | 0.94 (0.53) | 1.01 (0.52) | 0.86 (0.54) | <0.001 |  |
| PFNA at 28 y | 0.98 (0.60) | 1.14 (0.61) | 0.78 (0.49) | <0.001 |  | 0.94 (0.58) | 1.07 (0.61) | 0.77 (0.52) | <0.001 |  |
| PFDA at birth | 0.07 (0.06) | 0.07 (0.07) | 0.07 (0.06) | 0.61 |  | 0.07 (0.06) | 0.07 (0.07) | 0.07 (0.06) | 0.37 |  |
| PFDA at 7 y | 0.22 (0.15) | 0.21 (0.14) | 0.23 (0.17) | 0.08 |  | 0.22 (0.16) | 0.20 (0.14) | 0.24 (0.19) | 0.01 |  |
| PFDA at 14 y | 0.29 (0.17) | 0.32 (0.18) | 0.27 (0.15) | 0.24 |  | 0.28 (0.17) | 0.32 (0.19) | 0.27 (0.10) | 0.21 |  |
| PFDA at 22 y | 0.40 (0.25) | 0.42 (0.24) | 0.35 (0.24) | 0.003 |  | 0.39 (0.26) | 0.41 (0.24) | 0.37 (0.23) | 0.08 |  |
| PFDA at 28 y | 0.34 (0.25) | 0.40 (0.26) | 0.29 (0.19) | <0.001 |  | 0.34 (0.25) | 0.40 (0.24) | 0.30 (0.23) | <0.001 |  |
| ^a^ Sample sizes at birth and ages 7, 14, 22, and 28 years are (total: 388; male: 217; female: 171), (total: 280; male: 162; female: 118), (total: 177; male: 104; female: 73), (total: 361; male: 203; female: 158), and (total: 399; male: 220; female: 179), respectively.  ^b^ Sample sizes at birth and ages 7, 14, 22, and 28 years are (total: 276; male: 132; female: 144), (total: 122; male: 61; female: 61), (total: 127; male:65; female: 62), (total: 251; male:122; female: 129), and (total: 281; male: 133; female: 148), respectively.  ^c^ Wilcoxon rank-sum test used to evaluate whether PFAS concentrations differ between males and females.  ^d^ PFAS: per- and polyfluoroalkyl substance; PFOS: perfluorooctane sulfonate; PFOA: perflourooctanoate; PFHxS: perfluorohexanesulfonic acid; PFNA: perfluorononanoate; PFDA, perfluorodecanoate | | | | | | | | | | |

| Table S2. Unit change of serum anti-HAV concentrations (S/CO) at age 28 years per doubling of the cord blood PFAS concentrations and serum PFAS concentrations at ages 7, 14, 22, and 28 years. | | | | | | | | | | | | |
| --- | --- | --- | --- | --- | --- | --- | --- | --- | --- | --- | --- | --- |
| PFASs | Total cohort | | |  | Male | | |  | Female | | | P for interaction |
|  | n | % change | 95% CI |  | n | % change | 95% CI |  | n | % change | 95% CI |  |
| Cord blood PFAS concentrations | | | | | | | | | | | | |
| PFOS | 388 | 0.11 | -0.36, 0.59 |  | 217 | 0.75 | 0.13, 1.36 |  | 171 | -0.75 | -1.47, -0.03 | 0.006 |
| PFOA | 388 | 0.05 | -0.36, 0.46 |  | 217 | 0.49 | -0.02, 1.00 |  | 171 | -0.70 | -1.37, -0.03 | 0.01 |
| PFHxS | 388 | -0.06 | -0.32, 0.20 |  | 217 | 0.17 | -0.2, 0.53 |  | 171 | -0.28 | -0.64, 0.09 | 0.10 |
| PFNA | 388 | 0.02 | -0.25, 0.28 |  | 217 | 0.20 | -0.14, 0.54 |  | 171 | -0.27 | -0.69, 0.16 | 0.09 |
| PFDA | 388 | -0.11 | -0.44, 0.22 |  | 217 | 0.07 | -0.35, 0.49 |  | 171 | -0.38 | -0.91, 0.14 | 0.19 |
| PFAS concentrations at 7 years | | | | | | | | | | | | |
| PFOS | 280 | 0.21 | -0.54, 0.96 |  | 162 | -0.30 | -1.20, 0.59 |  | 118 | 1.36 | 0.03, 2.69 | 0.04 |
| PFOA | 280 | 0.10 | -0.52, 0.72 |  | 162 | -0.08 | -0.90, 0.75 |  | 118 | 0.33 | -0.60, 1.26 | 0.52 |
| PFHxS | 280 | 0.08 | -0.27, 0.42 |  | 162 | -0.06 | -0.51, 0.38 |  | 118 | 0.28 | -0.26, 0.83 | 0.34 |
| PFNA | 280 | 0.19 | -0.40, 0.77 |  | 162 | -0.18 | -0.93, 0.57 |  | 118 | 0.75 | -0.18, 1.68 | 0.13 |
| PFDA | 280 | 0.17 | -0.32, 0.66 |  | 162 | -0.19 | -0.83, 0.45 |  | 118 | 0.66 | -0.09, 1.41 | 0.09 |
| PFAS concentrations at 14 years | | | | | | | | | | | | |
| PFOS | 177 | -0.14 | -1.01, 0.74 |  | 104 | -0.26 | -1.22, 0.70 |  | 73 | 0.45 | -1.61, 2.51 | 0.54 |
| PFOA | 177 | -0.71 | -1.52, 0.09 |  | 104 | -0.99 | -1.92, -0.06 |  | 73 | 0.10 | -1.48, 1.68 | 0.24 |
| PFHxS | 177 | 0.05 | -0.50, 0.61 |  | 104 | 0.14 | -0.53, 0.80 |  | 73 | -0.14 | -1.14, 0.85 | 0.64 |
| PFNA | 177 | -0.11 | -0.77, 0.54 |  | 104 | 0.04 | -0.73, 0.82 |  | 73 | -0.5 | -1.71, 0.72 | 0.46 |
| PFDA | 177 | -0.06 | -0.71, 0.59 |  | 104 | 0.03 | -0.74, 0.80 |  | 73 | -0.29 | -1.50, 0.92 | 0.66 |
| PFAS concentrations at 22 years | | | | | | | | | | | | |
| PFOS | 361 | -0.10 | -0.63, 0.44 |  | 203 | -0.25 | -1.00, 0.50 |  | 158 | 0.06 | -0.71, 0.82 | 0.57 |
| PFOA | 361 | -0.06 | -0.48, 0.35 |  | 203 | -0.55 | -1.21, 0.12 |  | 158 | 0.24 | -0.29, 0.77 | 0.07 |
| PFHxS | 361 | -0.02 | -0.46, 0.42 |  | 203 | -0.21 | -0.87, 0.44 |  | 158 | 0.14 | -0.46, 0.74 | 0.44 |
| PFNA | 361 | 0.06 | -0.42, 0.55 |  | 203 | -0.2 | -0.96, 0.56 |  | 158 | 0.24 | -0.38, 0.86 | 0.38 |
| PFDA | 361 | 0.13 | -0.33, 0.60 |  | 203 | -0.08 | -0.79, 0.64 |  | 158 | 0.28 | -0.32, 0.89 | 0.45 |
| PFAS concentrations at 28 years | | | | | | | | | | | | |
| PFOS | 399 | -0.23 | -0.66, 0.21 |  | 220 | -0.19 | -0.85, 0.46 |  | 179 | -0.25 | -0.82, 0.32 | 0.90 |
| PFOA | 399 | -0.24 | -0.59, 0.10 |  | 220 | -0.34 | -0.96, 0.29 |  | 179 | -0.20 | -0.61, 0.20 | 0.72 |
| PFHxS | 399 | -0.09 | -0.55, 0.36 |  | 220 | 0.05 | -0.66, 0.76 |  | 179 | -0.19 | -0.78, 0.39 | 0.61 |
| PFNA | 399 | -0.12 | -0.56, 0.32 |  | 220 | -0.08 | -0.72, 0.56 |  | 179 | -0.16 | -0.77, 0.44 | 0.85 |
| PFDA | 399 | -0.05 | -0.42, 0.31 |  | 220 | -0.11 | -0.65, 0.42 |  | 179 | 0.00 | -0.49, 0.50 | 0.76 |
| ^a^ Models for total cohort were adjusted for sex.  ^b^ S/CO: signal-to-cutoff ratio; CI: confidence interval; PFAS: per- and polyfluoroalkyl substance; PFOS: perfluorooctane sulfonate; PFOA: perflourooctanoate; PFHxS: perfluorohexanesulfonic acid; PFNA: perfluorononanoate; PFDA, perfluorodecanoate. | | | | | | | | | | | | |

| Table S3. Percent change of serum anti-HBs concentrations at age 28 years per doubling of the cord blood PFAS concentrations and serum PFAS concentrations at ages 7, 14, 22, and 28 years. | | | | | | | | | | | | |
| --- | --- | --- | --- | --- | --- | --- | --- | --- | --- | --- | --- | --- |
| PFASs | Total cohort | | |  | Male | | |  | Female | | | P for interaction |
|  | n | % change | 95% CI |  | n | % change | 95% CI |  | n | % change | 95% CI |  |
| Cord blood PFAS concentrations | | | | | | | | | | | | |
| PFOS | 375 | -23.24 | -46.77, 10.69 |  | 210 | -19.17 | -50.75, 32.66 |  | 165 | -27.83 | -57.98, 23.95 | 0.76 |
| PFOA | 375 | -4.34 | -30.69, 32.02 |  | 210 | 4.55 | -31.08, 58.59 |  | 165 | -16.04 | -49.32, 39.11 | 0.51 |
| PFHxS | 375 | -2.85 | -20.43, 18.60 |  | 210 | 4.91 | -21.53, 40.26 |  | 165 | -9.25 | -30.95, 19.28 | 0.48 |
| PFNA | 375 | 7.52 | -12.42, 31.99 |  | 210 | 24.44 | -4.52, 62.17 |  | 165 | -13.06 | -36.83, 19.65 | 0.09 |
| PFDA | 375 | 5.75 | -17.63, 35.77 |  | 210 | 5.64 | -23.52, 45.93 |  | 165 | 5.91 | -28.46, 56.82 | 0.99 |
| PFAS concentrations at 7 years | | | | | | | | | | | | |
| PFOS | 269 | -4.65 | -45.84, 67.87 |  | 269 | -4.65 | -45.84, 67.87 |  | 113 | 226.53 | 14.90, 827.95 | 0.01 |
| PFOA | 269 | -9.39 | -43.40, 45.04 |  | 269 | -9.39 | -43.4, 45.04 |  | 113 | 68.31 | -16.19, 238 | 0.02 |
| PFHxS | 269 | -2.92 | -25.10, 25.83 |  | 269 | -2.92 | -25.1, 25.83 |  | 113 | 19.57 | -20.54, 79.92 | 0.20 |
| PFNA | 269 | 20.46 | -22.54, 87.32 |  | 269 | 20.46 | -22.54, 87.32 |  | 113 | 126.13 | 11.64, 358.03 | 0.03 |
| PFDA | 269 | 40.08 | -2.99, 102.28 |  | 269 | 40.08 | -2.99, 102.28 |  | 113 | 115.84 | 22.24, 281.10 | 0.05 |
| PFAS concentrations at 14 years | | | | | | | | | | | | |
| PFOS | 172 | 22.17 | -34.09, 126.46 |  | 100 | 23.39 | -37.41, 143.28 |  | 72 | 16.77 | -72.52, 396.24 | 0.95 |
| PFOA | 172 | -7.40 | -47.65, 63.81 |  | 100 | 3.41 | -46.55, 100.05 |  | 72 | -32.26 | -77.69, 105.67 | 0.52 |
| PFHxS | 172 | 10.62 | -25.11, 63.40 |  | 100 | 24.43 | -22.09, 98.71 |  | 72 | -14.40 | -57.12, 70.85 | 0.38 |
| PFNA | 172 | 23.24 | -22.05, 94.84 |  | 100 | 36.70 | -20.42, 134.82 |  | 72 | -4.21 | -58.80, 122.72 | 0.49 |
| PFDA | 172 | 22.36 | -22.31, 92.70 |  | 100 | 45.90 | -14.48, 148.90 |  | 72 | -20.63 | -65.65, 83.41 | 0.23 |
| PFAS concentrations at 22 years | | | | | | | | | | | | |
| PFOS | 350 | 15.26 | -22.88, 72.26 |  | 198 | 9.51 | -36.74, 89.58 |  | 152 | 22.22 | -32.07, 119.88 | 0.79 |
| PFOA | 350 | -21.24 | -42.20, 7.34 |  | 198 | -30.90 | -57.77, 13.06 |  | 152 | -14.26 | -42.31, 27.43 | 0.50 |
| PFHxS | 350 | -12.26 | -37.00, 22.19 |  | 198 | -18.15 | -49.57, 32.84 |  | 152 | -6.78 | -40.68, 46.50 | 0.70 |
| PFNA | 350 | 11.89 | -22.04, 60.58 |  | 198 | 27.20 | -27.27, 122.47 |  | 152 | 2.14 | -36.25, 63.64 | 0.56 |
| PFDA | 350 | 11.82 | -20.85, 57.97 |  | 198 | 22.00 | -27.99, 106.70 |  | 152 | 4.77 | -33.58, 65.24 | 0.67 |
| PFAS concentrations at 28 years | | | | | | | | | | | | |
| PFOS | 386 | 6.12 | -23.36, 46.93 |  | 213 | 26.21 | -22.33, 105.09 |  | 173 | -7.74 | -40.35, 42.70 | 0.35 |
| PFOA | 386 | -16.77 | -35.47, 7.35 |  | 213 | -9.21 | -43.16, 45.04 |  | 173 | -19.73 | -40.66, 8.60 | 0.67 |
| PFHxS | 386 | -16.79 | -40.65, 16.67 |  | 213 | 13.19 | -32.92, 91 |  | 173 | -33.00 | -56.81, 3.93 | 0.13 |
| PFNA | 386 | 11.88 | -19.31, 55.13 |  | 213 | 40.09 | -12.79, 125.01 |  | 173 | -8.47 | -41.51, 43.25 | 0.20 |
| PFDA | 386 | 19.49 | -8.82, 56.58 |  | 213 | 37.37 | -7.49, 104.00 |  | 173 | 5.85 | -26.78, 53.01 | 0.35 |
| ^a^ Models for total cohort were adjusted for sex.  ^b^ CI: confidence interval; PFAS: per- and polyfluoroalkyl substance; PFOS: perfluorooctane sulfonate; PFOA: perflourooctanoate; PFHxS: perfluorohexanesulfonic acid; PFNA: perfluorononanoate; PFDA, perfluorodecanoate. | | | | | | | | | | | | |

| Table S4. Percent change of the follow-up serum anti-diphtheria concentrations at age 28 years per doubling of the cord blood PFAS concentrations and serum PFAS concentrations at ages 7, 14, 22, and 28 years. | | | | | | | | | | | | |
| --- | --- | --- | --- | --- | --- | --- | --- | --- | --- | --- | --- | --- |
| PFASs | Total cohort | | |  | Male | | |  | Female | | | P for interaction |
|  | n | % change | 95% CI |  | n | % change | 95% CI |  | n | % change | 95% CI |  |
| Cord blood PFAS concentrations | | | | | | | | | | | | |
| PFOS | 276 | 28.26 | -5.7, 74.44 |  | 132 | 72.36 | 9.35, 171.67 |  | 144 | 0.78 | -33.18, 52.01 | 0.09 |
| PFOA | 276 | 28.14 | -0.38, 64.82 |  | 132 | 68.57 | 20.62, 135.57 |  | 144 | -8.52 | -36.88, 32.57 | 0.02 |
| PFHxS | 276 | 13.57 | -2.4, 32.15 |  | 132 | 14.72 | -10.98, 47.82 |  | 144 | 12.94 | -6.42, 36.32 | 0.92 |
| PFNA | 276 | -1.27 | -15.31, 15.09 |  | 132 | 9.44 | -11.61, 35.49 |  | 144 | -11.3 | -28.66, 10.28 | 0.18 |
| PFDA | 276 | 7.29 | -11.16, 29.58 |  | 132 | 16.16 | -10.63, 50.99 |  | 144 | -1.39 | -24.75, 29.22 | 0.4 |
| PFAS concentrations at 7 years | | | | | | | | | | | | |
| PFOS | 173 | 5.04 | -36.45, 73.59 |  | 89 | 26.87 | -31.52, 135.04 |  | 84 | -25.97 | -67.93, 70.9 | 0.31 |
| PFOA | 173 | -4.89 | -37.24, 44.11 |  | 89 | 32.26 | -26.13, 136.8 |  | 84 | -30.83 | -60.98, 22.62 | 0.12 |
| PFHxS | 173 | 1.96 | -18.98, 28.31 |  | 89 | 17.48 | -11.86, 56.59 |  | 84 | -18.74 | -43.42, 16.68 | 0.12 |
| PFNA | 173 | 35.02 | -7.9, 97.93 |  | 89 | 59.27 | -4.09, 164.49 |  | 84 | 9.76 | -37.77, 93.57 | 0.34 |
| PFDA | 173 | 37.89 | 1.77, 86.82 |  | 89 | 43.84 | -4.35, 116.33 |  | 84 | 30.99 | -16.46, 105.38 | 0.76 |
| PFAS concentrations at 14 years | | | | | | | | | | | | |
| PFOS | 127 | -3.50 | -42.87, 63.01 |  | 65 | -20.20 | -57.32, 49.23 |  | 62 | 46.43 | -42.05, 270.05 | 0.29 |
| PFOA | 127 | -11.60 | -47.55, 48.97 |  | 65 | -13.81 | -56.15, 69.40 |  | 62 | -8.33 | -59.21, 106.04 | 0.91 |
| PFHxS | 127 | -7.62 | -37.93, 37.48 |  | 65 | -7.20 | -47.17, 62.98 |  | 62 | -8.03 | -47.08, 59.84 | 0.98 |
| PFNA | 127 | 11.90 | -22.39, 61.36 |  | 65 | 0.98 | -36.39, 60.32 |  | 62 | 31.82 | -26.42, 136.16 | 0.48 |
| PFDA | 127 | -7.20 | -35.09, 32.68 |  | 65 | -20.50 | -47.44, 20.27 |  | 62 | 39.44 | -28.72, 172.77 | 0.16 |
| PFAS concentrations at 22 years | | | | | | | | | | | | |
| PFOS | 250 | 5.29 | -21.69, 41.56 |  | 121 | 23.83 | -19.77, 91.13 |  | 129 | -8.3 | -38.57, 36.89 | 0.32 |
| PFOA | 250 | 9.8 | -12.62, 37.96 |  | 121 | 16.95 | -21.84, 74.99 |  | 129 | 6.65 | -18.94, 40.32 | 0.71 |
| PFHxS | 250 | -8.44 | -27.27, 15.27 |  | 121 | 8.32 | -27.37, 61.54 |  | 129 | -15.68 | -36.26, 11.55 | 0.32 |
| PFNA | 250 | 45.39 | 11.66, 89.32 |  | 121 | 34.99 | -14.75, 113.78 |  | 129 | 50.73 | 9.39, 107.71 | 0.7 |
| PFDA | 250 | 34.94 | 4.92, 73.56 |  | 121 | 27.2 | -17.56, 96.25 |  | 129 | 39 | 2.23, 89 | 0.74 |
| PFAS concentrations at 28 years | | | | | | | | | | | | |
| PFOS | 281 | 6.91 | -14.26, 33.31 |  | 133 | 7.38 | -22.29, 48.38 |  | 148 | 6.51 | -21.08, 43.73 | 0.97 |
| PFOA | 281 | 23.56 | 3.65, 47.29 |  | 133 | 19.74 | -15.97, 70.63 |  | 148 | 24.83 | 2.02, 52.74 | 0.84 |
| PFHxS | 281 | 0.77 | -20.49, 27.7 |  | 133 | 6.14 | -30.04, 61.01 |  | 148 | -1.65 | -26.06, 30.84 | 0.77 |
| PFNA | 281 | 30.56 | 3.39, 64.86 |  | 133 | 25.02 | -13.64, 80.98 |  | 148 | 34.31 | -0.4, 81.12 | 0.77 |
| PFDA | 281 | 19.61 | -1.24, 44.86 |  | 133 | 12.81 | -16.16, 51.8 |  | 148 | 24.65 | -2.87, 59.95 | 0.61 |
| ^a^ Models for total cohort were adjusted for sex.  ^b^ CI: confidence interval; PFAS: per- and polyfluoroalkyl substance; PFOS: perfluorooctane sulfonate; PFOA: perflourooctanoate; PFHxS: perfluorohexanesulfonic acid; PFNA: perfluorononanoate; PFDA, perfluorodecanoate. | | | | | | | | | | | | |

| Table S5. Percent change of the follow-up serum anti-tetanus concentrations at age 28 years per doubling of the cord blood PFAS concentrations and serum PFAS concentrations at ages 7, 14, 22, and 28 years. | | | | | | | | | | | | |
| --- | --- | --- | --- | --- | --- | --- | --- | --- | --- | --- | --- | --- |
| PFASs | Total cohort | | |  | Male | | |  | Female | | | P for interaction |
|  | n | % change | 95% CI |  | n | % change | 95% CI |  | n | % change | 95% CI |  |
| Cord blood PFAS concentrations | | | | | | | | | | | | |
| PFOS | 276 | 2 | -20.24, 30.44 |  | 132 | -9.28 | -37.05, 30.74 |  | 144 | 12.23 | -19.32, 56.12 | 0.4 |
| PFOA | 276 | -2.57 | -20.38, 19.22 |  | 132 | -5.56 | -28, 23.88 |  | 144 | 1.23 | -25.07, 36.74 | 0.74 |
| PFHxS | 276 | 0.63 | -10.86, 13.6 |  | 132 | 0.74 | -17.78, 23.43 |  | 144 | 0.58 | -13.47, 16.91 | 0.99 |
| PFNA | 276 | -5.88 | -16.69, 6.33 |  | 132 | -4.78 | -19.72, 12.93 |  | 144 | -7.01 | -21.83, 10.63 | 0.85 |
| PFDA | 276 | -12.86 | -24.97, 1.2 |  | 132 | -8.79 | -25.99, 12.41 |  | 144 | -16.99 | -33.07, 2.97 | 0.54 |
| PFAS concentrations at 7 years | | | | | | | | | | | | |
| PFOS | 173 | 8.91 | -25.85, 59.95 |  | 89 | 10.92 | -31.08, 78.53 |  | 84 | 5.35 | -44.4, 99.61 | 0.9 |
| PFOA | 173 | 4.68 | -23.9, 43.99 |  | 89 | 1.23 | -35.9, 59.87 |  | 84 | 7.96 | -30.39, 67.41 | 0.84 |
| PFHxS | 173 | 3.23 | -13.22, 22.79 |  | 89 | 11.01 | -10.78, 38.13 |  | 84 | -8.27 | -30.54, 21.15 | 0.29 |
| PFNA | 173 | 14.64 | -14.68, 54.05 |  | 89 | 18.57 | -19.97, 75.65 |  | 84 | 9.94 | -29.05, 70.37 | 0.8 |
| PFDA | 173 | 3.22 | -18.46, 30.66 |  | 89 | 8.28 | -21.13, 48.66 |  | 84 | -2.6 | -31.26, 38.01 | 0.66 |
| PFAS concentrations at 14 years | | | | | | | | | | | | |
| PFOS | 127 | -19.44 | -48.36, 25.66 |  | 65 | -22.42 | -54.46, 32.16 |  | 62 | -12.49 | -60.26, 92.74 | 0.80 |
| PFOA | 127 | -0.77 | -36.35, 54.7 |  | 65 | -10.93 | -49.84, 58.17 |  | 62 | 15.88 | -41.77, 130.6 | 0.57 |
| PFHxS | 127 | -10.24 | -35.99, 25.87 |  | 65 | -1.37 | -39.02, 59.53 |  | 62 | -17.92 | -48.63, 31.14 | 0.59 |
| PFNA | 127 | -11.02 | -34.9, 21.6 |  | 65 | -13.85 | -42.01, 27.99 |  | 62 | -6.38 | -42.99, 53.72 | 0.80 |
| PFDA | 127 | -22.63 | -42.94, 4.91 |  | 65 | -14.28 | -39.71, 21.9 |  | 62 | -41.01 | -66.58, 4.12 | 0.27 |
| PFAS concentrations at 22 years | | | | | | | | | | | | |
| PFOS | 250 | -9.1 | -28.42, 15.44 |  | 121 | -6.99 | -34.48, 32.02 |  | 129 | -10.87 | -35.57, 23.3 | 0.86 |
| PFOA | 250 | -0.39 | -17.12, 19.72 |  | 121 | 12.77 | -18.45, 55.94 |  | 129 | -6 | -24.7, 17.32 | 0.36 |
| PFHxS | 250 | -3.47 | -19.88, 16.3 |  | 121 | 11.85 | -18.98, 54.4 |  | 129 | -10.25 | -28.45, 12.57 | 0.27 |
| PFNA | 250 | -1.27 | -20.54, 22.66 |  | 121 | 0.67 | -30.93, 46.72 |  | 129 | -2.22 | -24.95, 27.41 | 0.9 |
| PFDA | 250 | -5.24 | -22.88, 16.44 |  | 121 | -7.57 | -35.07, 31.59 |  | 129 | -4.03 | -25.44, 23.53 | 0.87 |
| PFAS concentrations at 28 years | | | | | | | | | | | | |
| PFOS | 281 | -2.1 | -17.77, 16.56 |  | 133 | -9.3 | -29.82, 17.21 |  | 148 | 4.54 | -17.57, 32.57 | 0.43 |
| PFOA | 281 | 3.1 | -10.42, 18.66 |  | 133 | 10.11 | -17.16, 46.35 |  | 148 | 0.92 | -14.13, 18.62 | 0.6 |
| PFHxS | 281 | -5.62 | -21.67, 13.72 |  | 133 | -3.55 | -30.61, 34.07 |  | 148 | -6.58 | -25.48, 17.1 | 0.88 |
| PFNA | 281 | -5.19 | -21.29, 14.21 |  | 133 | -3.77 | -28.41, 29.35 |  | 148 | -6.11 | -26.12, 19.34 | 0.9 |
| PFDA | 281 | -5.29 | -18.66, 10.29 |  | 133 | -9.86 | -28.84, 14.19 |  | 148 | -1.89 | -19.61, 19.75 | 0.59 |
| ^a^ Models for total cohort were adjusted for sex.  ^b^ CI: confidence interval; PFAS: per- and polyfluoroalkyl substance; PFOS: perfluorooctane sulfonate; PFOA: perflourooctanoate; PFHxS: perfluorohexanesulfonic acid; PFNA: perfluorononanoate; PFDA, perfluorodecanoate. | | | | | | | | | | | | |

| Table S6. Sample size of the matched datasets for the Rubin Causal analysis. | | | |
| --- | --- | --- | --- |
| Time point | All | Male | Female |
| Birth | 280 | 159 | 121 |
| 7 years | 176 | 96 | 80 |
| 14 years | 114 | 64 | 50 |
| 22 years | 250 | 144 | 106 |
| 28 years | 274 | 150 | 124 |
